# Traditional Chinese Medicine for preventing influenza: a systematic review and meta-analysis

**DOI:** 10.3389/fmed.2026.1736574

**Published:** 2026-04-23

**Authors:** Haowen Huang, Yi Yuan, Huanxin Li, Qiushi Yu, Lijuan Xue, Bofei Meng, Qifei Li, Siyi Kui, Anargul Tudi, Xiaoqing Liu, Duoduo Li

**Affiliations:** 1 Dongzhimen Hospital, Beijing University of Chinese Medicine, Beijing, China; 2 Centre for Evidence-Based Chinese Medicine, Beijing University of Chinese Medicine, Beijing, China; 3 School of Traditional Chinese Medicine, Beijing University of Chinese Medicine, Beijing, China

**Keywords:** influenza, traditional Chinese medicine, prevention, meta-analysis, quantitative synthesis

## Abstract

**Background:**

Influenza is an acute infectious respiratory disease causing substantial public health and economic burdens. Multiple clinical studies have investigated traditional Chinese medicine (TCM) for influenza prevention.

**Objective:**

To assess the effectiveness and safety of TCM preventive interventions against influenza through direct comparisons.

**Methods:**

We systematically searched PubMed, EMbase, CENTRAL, Web of Science, CNKI, WanFang, CQVIP, and Sinomed up to August 14, 2025. A two-stage screening process (title/abstract screening followed by full-text screening) was implemented. Data extraction utilized predefined templates, followed by methodological quality assessment, statistical analysis, and GRADE assessment. All procedures were independently conducted by two investigators with cross-verification, with discrepancies resolved by a third party.

**Results:**

19 studies involving 55,183 healthy participants were included, comprising special populations of 600 pregnant women, 226 children, and 31,889 school populations, 3,238 community residents, and 210 military residents. Compared with no intervention, TCM demonstrated potential reduction in influenza incidence (RR = 0.23, 95% CI 0.12–0.43, *P* <0.00001, *I*^2^ = 94%, 13 studies, 38,463 participants) and influenza-like illness (RR = 0.38, 95% CI 0.23–0.63, 6 studies, 10,754 participants), with no statistically significant difference in adverse event rates (RR = 5.00, 95% CI 0.59–42.37, *P* = 0.14, *I*^2^ = 0%, 6 studies, 7,486 participants).

**Conclusion:**

TCM interventions may exhibit potential efficacy for influenza prevention, though the confidence in this conclusion is limited by the methodological limitations of included studies involving healthy populations. Very low certainty of the evidence suggests that TCM might reduce influenza and influenza-like illness incidence with potential safety profiles. We did not identify high certainty of the evidence regarding the effectiveness and safety of any preventive measures. Future research should employ rigorous methodologies, standardized influenza diagnostic criteria, and core outcome measures specific to influenza prevention.

**Systematic review registration:**

[https://www.crd.york.ac.uk/PROSPERO/view/CRD42024506941], identifier [CRD42024506941]

## 1 Introduction

Influenza is an acute respiratory infectious disease caused by the influenza virus. Influenza A and B viruses circulate seasonally each year, with influenza A virus having the potential to cause a global pandemic (1). Based on differences in nuclear and matrix proteins, it can be categorized into four types: A, B, C, and D. Currently, the primary human infections are the H1N1 and H3N2 subtypes of influenza A virus, as well as the Victoria and Yamagata lineages of influenza B virus (1). Influenza is more prevalent during the winter and spring seasons, with an incubation period typically ranging from 1 to 4 days. Its primary clinical manifestations include sudden onset, high fever, headache, and muscle pain, which may be accompanied by symptoms such as chills, sore throat, dry cough, fatigue, decreased appetite, and diarrhea (2). In severe cases, it can lead to multiple complications including pneumonia, and even death (2).

From an epidemiological perspective, the World Health Organization estimates that influenza results in 3–5 million severe cases and between 290,000 and 650,000 respiratory disease-related deaths globally each year (3). Using data from the National Influenza-Like Illness Surveillance Sentinel Hospital, it is estimated that approximately 3.4 million cases in China receive medical treatment for influenza-like illnesses annually (4), with an average of roughly 88,100 deaths attributable to influenza-related respiratory diseases each year, accounting for 8.2% of all respiratory disease deaths (5). From an economic standpoint, the average annual economic burden associated with seasonal influenza in the United States is estimated to be 11.2 billion dollars, with 8 billion dollars in indirect costs. In Europe, the total annual cost of influenza is estimated to range from 6 to 14 billion euros (6). In conclusion, influenza has imposed a significant socio-economic burden on the global healthcare system.

At present, the most common flu prevention measure targets high-risk populations through vaccination, but research results (7) indicate that its preventive effect is not ideal, with an average protective efficacy of about 60%, and this effect gradually diminishes over time. As neuraminidase inhibitors, oseltamivir and zanamivir can effectively prevent influenza. The results of a randomized, controlled, double-blind trial involving healthy adults who had not been vaccinated demonstrated that oseltamivir has an effectiveness rate of 74% in preventing influenza (8). Furthermore, zanamivir is effective in preventing both influenza A and influenza B (9, 10). When administered as a prophylactic drug, neuraminidase inhibitors can selectively inhibit the activity of the neuraminidase on the surface of respiratory viruses, thereby preventing the replication and release of progeny virus particles in human cells. There are studies (11) suggesting that neuraminidase inhibitors can prevent viruses from entering epithelial cells and subsequently invading the respiratory tract, thus playing a protective role in influenza prevention. An overview (12) conducted by our team previously showed that amantadine, garlic and six different vaccines were beneficial in reducing the incidence of influenza compared to a placebo, while oseltamivir, zanamivir, Ganmao Capsules, echinacea and three other vaccines might be beneficial. The indirect comparison results from the Network meta-analysis (12) revealed that Ganmao Capsules had the highest effectiveness in preventing flu, followed by amantadine, garlic, and all types of vaccines. Although these results suggested that TCM may have some effectiveness in preventing influenza, they were constrained by the quality of the methodology and the quantity of relevant evidence, and therefore cannot provide high certainty of the evidence for clinical application.

The results of a clinical controlled trial indicated that the combination of Sangju Yin and Yupingfeng San is beneficial for improving influenza-like symptoms and preventing severe acute respiratory syndrome (13). Maxingshigan Decoction has been shown to augment the expression of IL-2 and IL-4 in mice infected with influenza A virus, elevate the secretion of IFN-γ, and regulate the ratio of T-cell subsets (14, 15), suggesting that it may prevent influenza by stimulating the immune system (16). Catechin extract exhibits a significant inhibitory effect on cells infected with influenza A virus and can inhibit the proliferation of the virus in chicken embryos (17). Pretreatment with mixed polysaccharides extracted from TCM can enhance resistance to H1N1 virus infection and diminish both the lung index and lung injury (18). Furthermore, TCM can inhibit pathogen infection in the respiratory tract and modulate the function of the immune system, thereby contributing to the alleviation of infection (16). Although numerous clinical studies have been conducted on the prevention of influenza with TCM, there is a lack of summarized evidence between various TCM prevention measures.

This review summarizes and analyzes the effectiveness and safety of various TCM preventative measures for influenza through direct comparisons, utilizing systematic review and meta-analysis methods. The aim was to provide evidence-based medical evidence for clinical practice and to assist relevant decision-makers in developing guidelines.

## 2 Methods

The meta-analysis was registered in PROSPERO (CRD42024506941) and reporting standard followed the PRISMA 2020 statement (19).

### 2.1 Type of study

We included randomized controlled trials, non-randomized controlled trials (the controlled trial did not mention that they were randomized), and cohort studies investigating various TCM interventions for influenza prevention. Throughout the study, no included studies had missing data or exhibited obvious statistical errors, thus contacting the authors of relevant studies to obtain the original data was unnecessary.

### 2.2 Type of participants

Influenza is defined by the presence of clinical symptoms such as high fever (39–40°C), chills, headache, myalgia, fatigue, and anorexia, accompanied by laboratory-confirmed influenza virus infection. We included healthy participants without influenza, though they may have had underlying chronic diseases. There were no restrictions on the participants’ gender, age, race or comorbid medical conditions.

### 2.3 Type of interventions

Eligible interventions (exposure factors) encompassed the oral or topical application of TCM decoctions (It refers to the liquid dosage form created by mixing Chinese medicine pieces and soaking them in water, removing the residue, and extracting the juice. For example, Sangju decoction, etc.), proprietary Chinese medicines (Chinese medicinal materials are processed into Chinese medicinal products with certain dosage forms according to prescribed prescriptions and preparation techniques. Common dosage forms include injections, oral liquid dosage forms (such as solutions, suspensions, emulsions), oral solid dosage forms (such as powders, capsules, tablets, pills), etc. Examples include Lianhua Qingwen granules and Lanqin oral liquid), TCM sachets (It generally refers to the cloth pouch containing Chinese medicinal materials that is hung or placed in the room), TCM patches (They refer to the application of Chinese herbal preparations onto the skin, acupoints, and diseased areas for external treatment in TCM, acupuncture, moxibustion, cupping, massage, etc.) These interventions could be implemented either as standalone treatments or in combination with other TCM therapies.

Control measures comprised various comparator interventions including placebo, no-treatment control (blank control), standard medical care, and active controls (such as western medicine or alternative TCM therapies, etc.).

### 2.4 Type of outcome

Relevant studies must report at least one of the following outcome measures:

Primary outcome:

- lIncidence of influenza (defined as the presence of clinical symptoms including high fever (39–40°C), chills, headache, myalgia, fatigue, and anorexia, with laboratory-confirmed influenza virus infection).

Secondary outcome:

- lIncidence of influenza-like illness (defined as clinical manifestations resembling influenza, including fever (> 37.5°C), chills, fatigue, headache, myalgia, respiratory catarrhal symptoms, nasal congestion, and rhinorrhea).
- lHospitalization rate.

Safety outcome:

- lAdverse events.

### 2.5 Search strategy

The following literature databases were systematically searched: PubMed, EMbase, CENTRAL (Cochrane Central Registry of Controlled Trials), Web of Science, China National Knowledge Infrastructure (CNKI), Wanfang Data, CQVIP, and Sinomed. The search time limited on August 14, 2025. Relevant databases utilized MeSH terms in combination with title and abstract search terms. We appropriately adjusted the retrieval strategy according to the search syntax of each database. Additionally, we supplemented the database search with manual searching of the references. See Supplementary material 1 for the retrieval strategy.

### 2.6 Study selection and data extraction

Two reviewers (HHW, YQS) used the literature management software NoteExpress (version 3.2.0; Beijing Aegean Lezhi Technology Co., Ltd.) to remove duplicates and screen the literature based on the inclusion and exclusion criteria. The screening process was divided into two stages: title and abstract screening and full-text screening, which were conducted back-to-back and cross-checked by the two reviewers. After confirming the included studies, the two reviewers used a standard data extraction form to extract data. In case of disagreements in the above stages, they referred to a third party (YY). For each included study, we extracted the following information:

- Basic information: such as title, author, publication date, sample size;
- Participants information: such as demographic data, age, sex, chronic disease, race;
- Details of interventions (exposure factors) and comparisons: name of intervention (exposure factors), dosage, course of treatment, combined intervention therapies;
- Outcome indicators: definition, result.

### 2.7 Methodological quality assessment

Two reviewers (HHW, LHX) conducted the methodological quality assessment according to the Cochrane Handbook (20). We utilized the Cochrane Risk of Bias tool for this assessment, which encompasses the following five domains: bias in the randomization process, bias due to intervention measures, bias arising from outcome data, bias in outcome measurement, and selective reporting of results.

For non-randomized controlled trials, we assessed the risk of bias using the ROBINS-I tool, which involved seven dimensions: bias due to confounding, bias in selection of participants into the study, bias in classification of interventions, bias due to deviations from intended interventions, bias due to missing data, bias in measurement of outcomes, bias in selection of the reported result.

For cohort studies, we employed the Newcastle-Ottawa Scale (NOS) for methodological quality evaluation. The NOS evaluation comprises three components: selection, comparability, and outcome measurement of cohorts. In the event of any disagreement during the assessment process, it will be resolved by a third party (YY).

### 2.8 Data analysis

Quantitative synthesis was completed using RevMan software. We chose the risk ratio (RR) as the effect size and utilized the Mantel-Haenszel random-effects method to analyze dichotomous data. For continuous data, we selected the standardized mean difference (SMD) as the effect size and applied the Inverse Variance random-effects method for analysis. Heterogeneity was assessed using the Chi-square test and the *I*^2^ statistic. The following *I*^2^ thresholds were used: < 40% was defined as indicating that heterogeneity might not be important; an *I*^2^ value in the range of 40–60% was considered to represent a moderate degree of heterogeneity; and > 60% was defined as indicating substantial heterogeneity. Since more than 10 studies were included in the pooled analysis for the primary outcome (incorporating independent data points from repeated measurements within the same studies), we performed Egger’s test exclusively for the primary outcome using STATA software (version 15.0; STATA Corporation, College Station, TX, United States). We assessed publication bias among the included studies through funnel plots and Egger’s test.

To comprehensively capture available evidence while maintaining methodological rigor, we included randomized controlled trials (RCTs), non-randomized controlled trials (non-RCTs), and cohort studies. Given the inherent differences in study design and risk of bias, we adopted a stratified approach to data synthesis: (1) RCTs and non-RCTs were pooled together in subgroup analyses by intervention type and age group using random-effects models, as they share a controlled trial design; (2) The single eligible cohort study was analyzed separately and not combined with controlled trials to avoid confounding by design-related bias. All forest plots and figure legends clearly indicate the design of included studies.

In the review registration, we planned to conduct a network meta-analysis. However, due to the extremely high heterogeneity among the studies and the fact that the network did not form any closed loops, the results obtained had very low reliability and validity. Therefore, we did not conduct a network meta-analysis on the data.

Due to the limited number of included studies, we were unable to conduct sensitivity analyses or subgroup analyses by influenza subtypes. However, we performed subgroup analyses based on different study designs (cohort studies, randomized controlled trials, and non-randomized controlled trials) as well as different age groups (children and adolescents: 0–19 years old; adults: 20–59 years old; elderly: 60 years and above).

We used GRADEpro GDT for GRADE assessment. The GRADE assessment has been used to appraise the certainty of the evidence for direct comparisons (21). Evidence assessment results for the primary and secondary outcomes were reported using the “Summary of findings” tables.

### 2.9 Ethics and dissemination

Among the 19 original studies included in this research, 7 studies mentioned that they had undergone ethical review, while the remaining 12 studies did not mention any ethical situation. However, since this review utilized published clinical study data and did not involve specific patient personal information. Consequently, ethical review board approval was not required for this study, and no ethical issues were identified. The results of this review were published in a peer-reviewed journal, and any researcher or reader can contact us via email to obtain the relevant data and software code for this review.

## 3 Results

### 3.1 Search result

**FIGURE 1:**
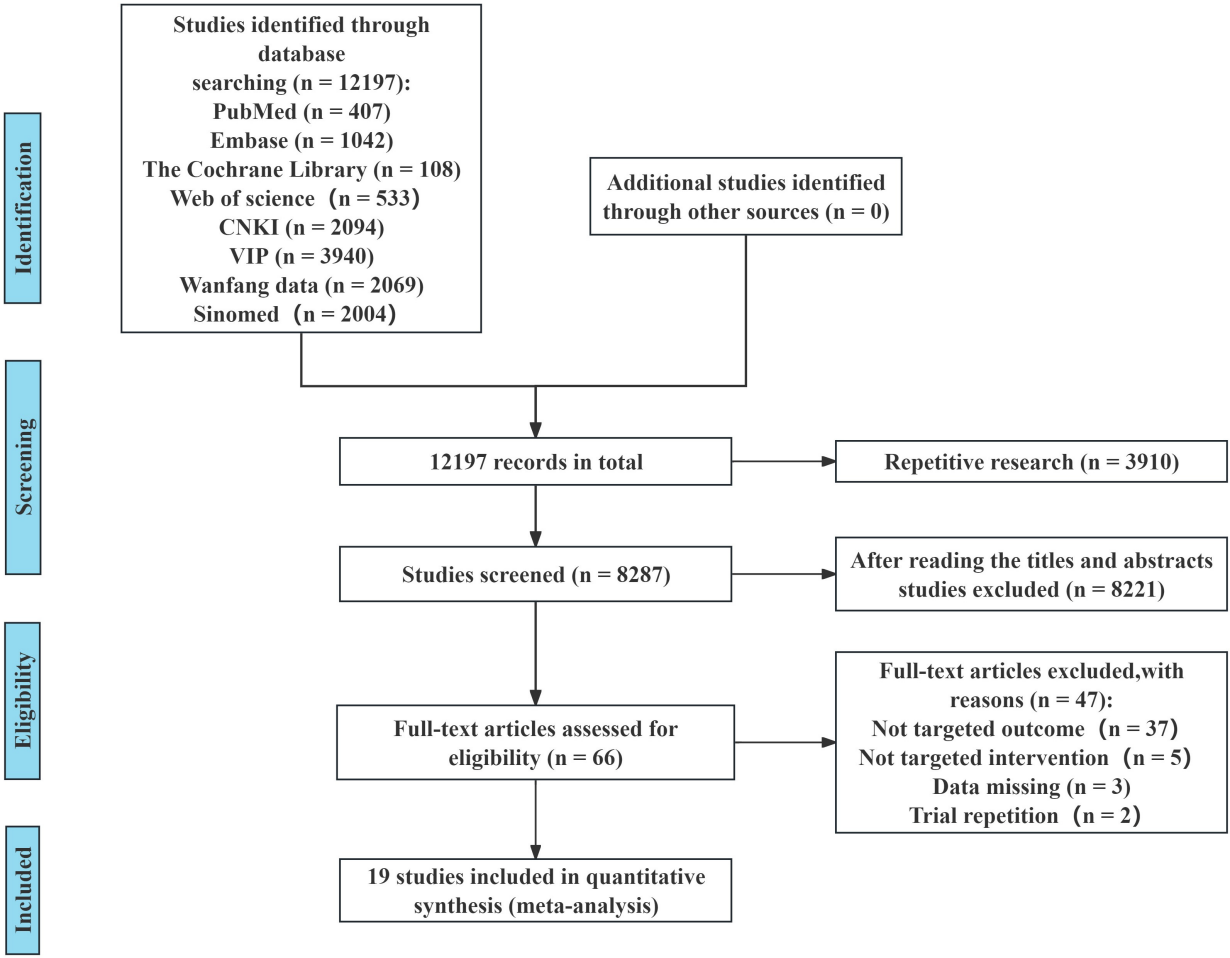
Flowchart of study selection.

Our systematic search across relevant databases identified 12,197 citations through August 14, 2025. After automated duplicate removal, 8,287 citations remained. During the title and abstract screening phase, 8,221 citations were excluded due to irrelevance to influenza or TCM, mismatched study types, or ineligible populations. In the subsequent full-text screening phase, 47 studies were excluded for reasons such as mismatched outcome measures, non-comparative study designs, incomplete data, or duplicate trial content. Ultimately, 19 studies were included in the analysis for this review. All included studies were published in Chinese (see Figure 1).

### 3.2 Characteristics of included studies

This review included 19 studies comprising15 randomized controlled trials, 3 non-randomized controlled trials, and 1 cohort study (Table 1).

**TABLE 1:** Characteristics of included trials on Traditional Chinese Medicine for preventing influenza.

Study ID	Study design	Sample size	Gender (M/F)	Age(years, average(mean ± SD) or range)	Treatment	Control	Course of the treatment	Outcome
Chen and Luo (28)	RCT	T:112 C:114	120/106	T: range 3–6, average 4.8	Sanfu plaster(3 hours external)	No treatment	3 years (3 days per year)	➀
Chen (29)	RCT	T:100 C:100	T:35/65 C:33/67	T: range 19–68, average (47.16 ± 12.89) C: range 20–60, average (46.89 ± 12.44)	Epidemic prevention sachets of traditional Chinese medicine (>tid external)	No treatment	1 month	➀ ➃ ➄
Deng (26)	RCT	T:335 C:315	T:127/208 C:114/201	T: range 11–92, average 44.10 ± 15.33 C: range 9-85, average 40.87 ± 15.09	Pihui Jiedu Prescription (bid, oral)	No treatment	15 days	➀ ➁ ➂ ➃
Li (35)	RCT	T:105 C:105	T:60/45 C:62/43	T: range 11–75, average 36.5 ± 1.96 C: range 8–73, average 34.2 ± 1.48	Healthy Qi—invigorating Anti-Inflammatory Formula (bid, oral)	No treatment	3 years (Every spring and autumn season, twice a season, take for three consecutive days each time)	➀ ➃
Li et al. (31)	RCT	T:80 C:80	T:22/58 C:25/55	T: range 36. 73 ± 11. 23 C: range 35. 35 ± 11. 53	(>tid external)	No treatment	2 months	➁ ➃ ➄
Li et al. (25)	RCT	T:31 C:32	T:22/9 C:25/7	T: range 51–68, average 62. 00 ± 3. 20 C: range 55–67, average 63. 00 ± 1. 89	Ancient recipe scented sachet(external)	No treatment	3 months	➀
Liu (39)	nRCT	T: 4,146/1,9896/2,669 C:1,382	NR	NR	Self-made Chinese medicinal formulae 1 (qd, oral)	No treatment	5 days	➀
Liu et al. (22)	RCT	T:28 C:25	T:6/22 C:5/20	T: range 30.5 ± 5.3 C: range 31.4 ± 4.7	Preventive prescription for H1N1 influenza(oral)	No treatment	7 Days	➀
Luo (37)	nRCT	T:1800 C:2200	T:1,200/600 C:1,500/700	NR	Preventing influenza cold tea (biw oral) and disinfecting classrooms (qd external) and dormitories (qw external) with mugwort fumigation and steaming	No treatment	1 month	➀
Qiu et al. (23)	RCT	T:300 C:300	T:0/300 C:0/300	T: range 19–37 C: range 19–35	Baopregnancy-kangdufang granule(1 dose every 5 days, oral)	No treatment	until 28 weeks of pregnancy	➀ ➁
Song et al. (27)	RCT	T:100 C:100	T:55/45 C:43/57	T: range 12–59, average (27.13 ± 14.49) C: range 14–62, average (25.63 ± 14.17)	Qingjie Fanggan granules (bid, oral)	No treatment	3days	➀
Su et al. (33)	RCT	T: Adult group/Elderly group: 815/755 C: Adult group/Elderly group: 872/636	T: Adult group:286/529 Elderly group:309/446 C: Adult group:320/552 Elderly group:279/357	T: Adult group: average (51.18 ± 10.5) Elderly group: average (72.88 ± 4.99) C: Adult group: average (51.05 ± 10.63) Elderly group: average (72.85 ± 5.04)	Preventive decoction of adults or the elderly (bid, oral)	No treatment	3 months (1 week every month)	➁
Su (36)	nRCT	T:89 C:67	T:27/62 C:28/39	T: range 21–71, average (38 ± 16.08) C: range 21–69, average(30 ± 10.39)	Influenza Preventive Formulae (bid, oral)	No treatment	5 days	➀
Wang (24)	RCT	T:5,128 C:5,128	T: 2,163/2,965 C:2,481/2,647	T: range 24–69, average (40.43 ± 11.84) C: range 21-68, average(42.36 ± 12.96)	Fuzheng Gubiao granules (bid, oral)	No treatment	7 days	➀
Wang and Meng (30)	RCT	T:45 C:40	50/35	T: average 32.5	Xinhuang Pian(bid, oral)	Indomethacin	2 weeks	➄
Zan et al. (40)	CS	T:384 C:384	T:154/230 C:154/230	T: 40(33, 52) C: 40(33, 50)*	Qinggandong Decoction(qd/bid/tid, oral)	No treatment	7-90days	➀ ➃
Zhang (38)	RCT	T:28/33/26 C:32	NR	NR	➀ Lianhua Qingwen capsule; ➁ Lianhua Qingwen capsule+antivirus granule; ➂ antivirus granule	No treatment	3days	➀
Zhang et al. (34)	RCT	T:1633 C:1633	T:862/771 C:862/771	T:12.7 ± 3⋅2 C:13.1 ± 3⋅4	Self-made Chinese medicinal formulae 2(bid, oral)	No treatment	2 months	➁ ➃
Zhao (32)	RCT	T:1500 C:1500	T:786/714 C:791/709	T: range 7-16, average(13.48 ± 2.56) C: range 7-16, average(13.20 ± 2.37)	Self-made Chinese medicinal formulae 3(bid, oral)	No treatment	NR	➁ ➃

RCT, randomized controlled trials; nRCT, non-randomized controlled trials; CS, Cohort studies; T, Treatment group; C, Control group; Qd, once a day; Qw, once a week; Bid, twice a day; Tid, three times a day. ➀ Incidence of influenza; ➁ Incidence of influenza-like illness; ➂ Hospitalization rate; ➃ Adverse events; ➄ Symptoms of influenza-like illness.

*M(P25, P75).

Among the 18 randomized controlled trials and non-randomized controlled trials, 15 studies (22–36) reported the age of participants, while 3 studies (37–39) did not provide age information. In the studies reporting age ranges, the minimum age was 3 years, and the maximum age was 92 years. Two studies focused on specific age groups: one study (33) categorized participants into an adult group (18–65 years) and an elderly group (> 65 years), while another study (28) exclusively enrolled children (3–6 years). 16 studies (22–37) reported gender distributions, totaling 12,698 males and 13,505 females across 26,203 participants. Notably, one study (23) specifically targeted pregnant women. The treatment duration ranged from 3 days to 3 years (with treatment administered for 3 days annually). 13 studies measured influenza incidence (22–29, 35–39), while 6 studies (23, 26, 31–34) reported the incidence of influenza-like illness. Hospitalization rates were assessed in only 1 study (26), and 6 studies (26, 29, 31, 32, 34, 35) reported adverse events.

19 TCM interventions were evaluated (see Supplementary material 2 for the details on the formulations and compositions). Only one study (30) used indomethacin as a control, while the remaining studies employed blank controls.

### 3.3 Methodological quality assessment

**FIGURE 2:**
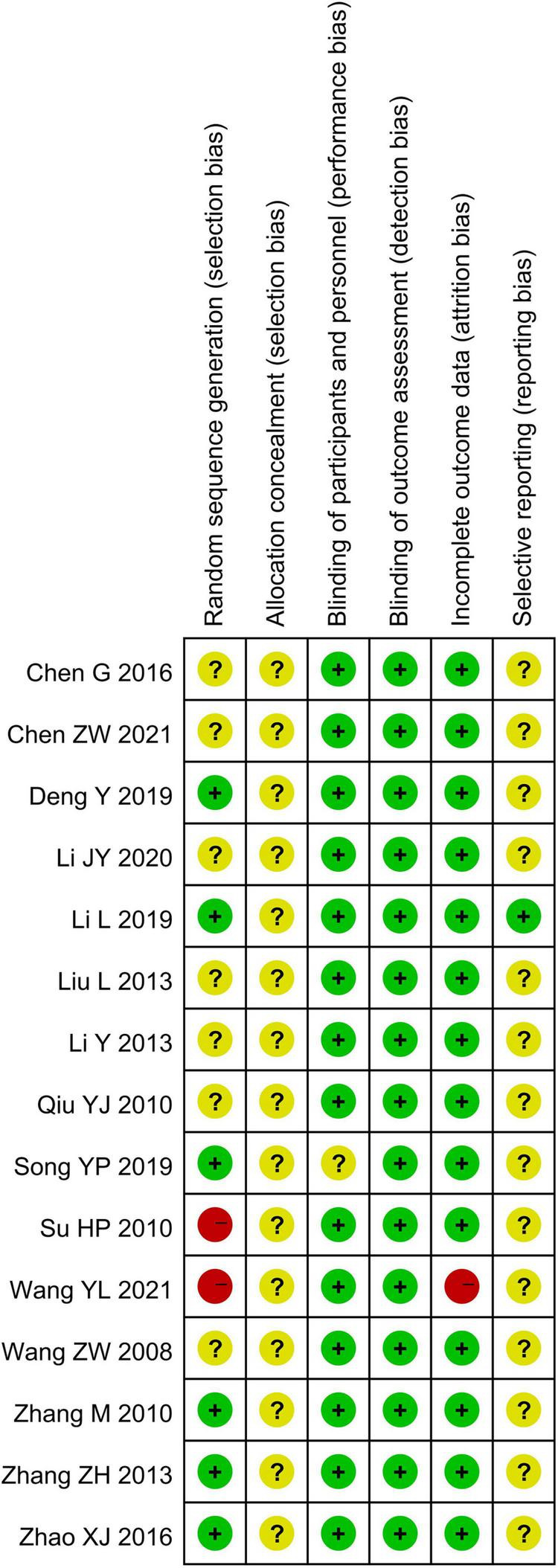
Risk of bias graph.

**FIGURE 3:**
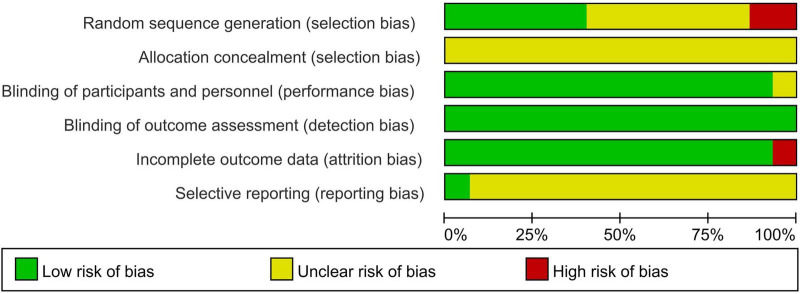
Risk of bias summary.

The methodological quality of all included randomized controlled trials was evaluated using the Cochrane Risk of Bias original tool (see Figures 2, 3).

Random sequence generation (selection bias): Six studies demonstrated low risk, including three studies (26, 27, 31) that utilized random number table, two (32, 34) employed cluster randomization, and one (38) applying a randomized lottery method. Two studies (24, 33) were rated as high risk due to non-random allocation based on the consultation order and patient preference, respectively. The remaining studies lacked randomization details and were rated as unclear risk.

Allocation concealment (selection bias): No studies described allocation concealment procedures, resulting in an unclear risk rating for all 15 included studies (22–35, 38).

Blinding of participants and personnel (performance bias): One study (27) reported placebo use but provided insufficient placebo details, resulting in an unclear risk rating. The remaining studies (22–26, 28–35, 38) utilized non-placebo controls. However, lack of blinding was deemed unlikely to influence objective outcome measures. Thus, these studies were rated as low risk of bias.

Blinding of outcome assessment (detection bias): All studies involved objective outcome measures minimally affected by outcome assessor blinding. Therefore, these studies were rated as low risk of bias.

Incomplete outcome data (attrition bias): One study (24) reported 63 participant withdrawals without clarifying data handling methods, resulting in a high-risk rating. The remaining 14 studies (22, 23, 25–35, 38) reported complete follow-up with no missing data, achieving a low-risk rating.

Selective reporting (reporting bias): Only one study (31) was prospectively registered on the Chinese Clinical Trial Registry, achieving a low-risk rating. Other studies lacked publicly accessible protocols, resulting in an unclear risk rating.

The three non-randomized controlled trials in our analysis were assessed using the ROBINS-I tool, receiving an overall moderate risk of bias assessment. In the first domain (Bias due to confounding), the study demonstrated moderate risk due to potential confounding variables affecting intervention outcomes. All six remaining bias domains were rated as low risk (see Supplementary material 3).

The included cohort study (40) was assessed using the Newcastle-Ottawa Scale (NOS). In the selection domain, the study demonstrated partial representation of the general exposed population, selected the non-exposed group in a manner consistent with the exposed group, ensured that no disease was present at baseline, objectively recorded or professionally assessed exposure factors, and confirmed that the outcome of interest was not present at the start of the study. Regarding comparability, the study controlled for key confounding factors, with no significant differences between study and control groups in major factors. In the outcome domain, results were derived from pathogen detection, the follow-up duration was sufficient, and all participants completed follow-up. Consequently, the cohort study achieved an NOS score of 8.5, demonstrating high methodological quality (41) (see Supplementary material 3).

#### 3.3.1 Diagnostic methods for influenza and influenza-like illness

Among the 19 included studies, diagnostic methods varied considerably. Six studies reported influenza incidence using laboratory-confirmed methods: two used polymerase chain reaction or viral culture (27, 39), and four used rapid antigen tests (23, 26, 36, 38). Eight studies reported influenza-like illness incidence based on clinical definitions: three defined it as fever with cough or sore throat (29, 31, 37), two adopted the ICHPPC-2-Defined criteria requiring fever of at least 38.5 degrees Celsius with at least four symptoms (32, 34), one used fever with sore throat combined with epidemiological history (33), one used trial termination criteria (26), and one diagnosed based on clinical symptoms during influenza season (28). Three studies relied solely on clinical observation without laboratory confirmation (22, 25, 40). Two studies had insufficient diagnostic information (30, 35). This heterogeneity in diagnostic methods may contribute to the high statistical heterogeneity observed and should be considered when interpreting the results.

### 3.4 Direct comparison results

#### 3.4.1 The incidence of influenza

**FIGURE 4:**
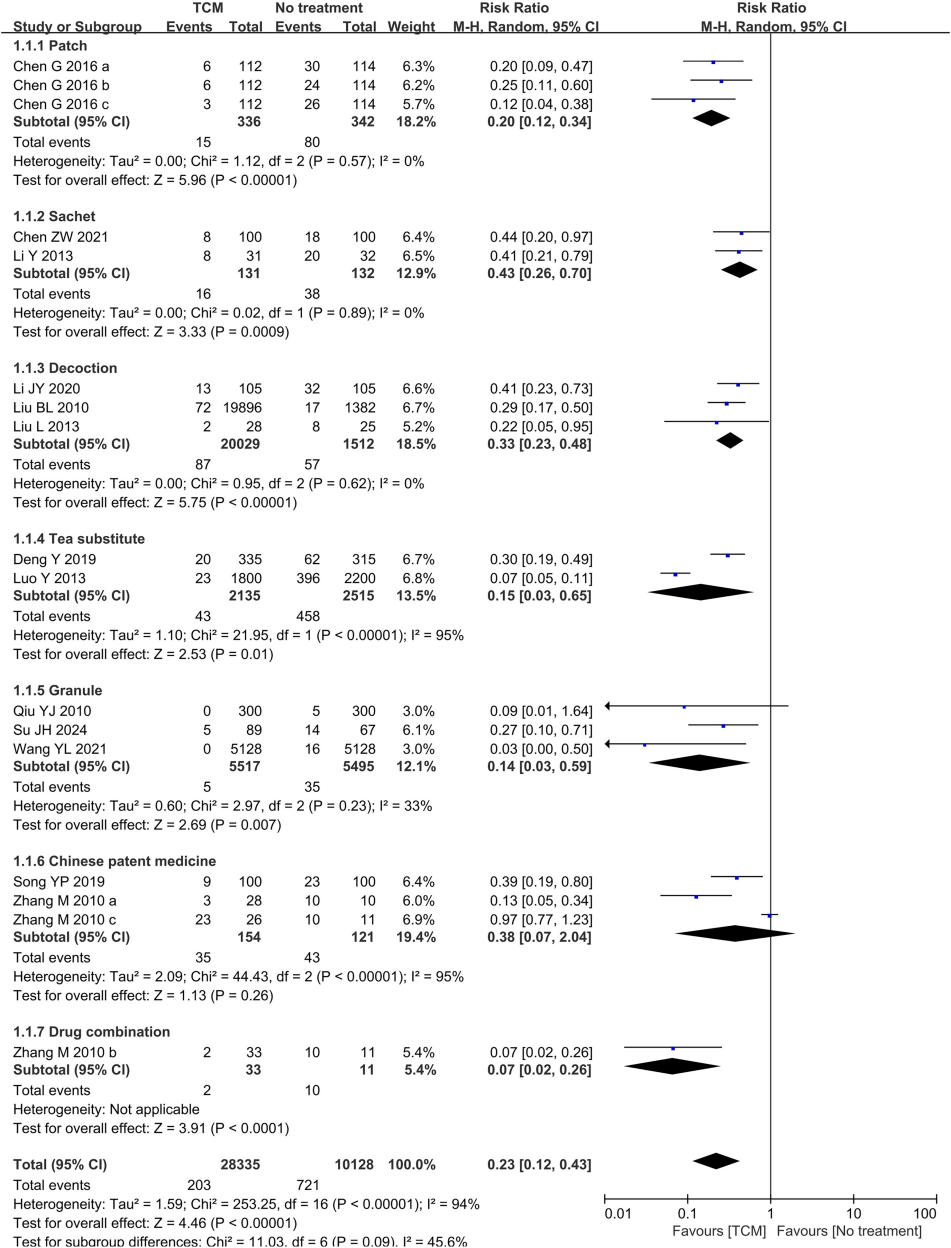
Forest plot of influenza incidence [subgroup by intervention type: Patch: Sanfu plaster (28); Sachet: epidemic prevention sachets of traditional Chinese medicine (29), Ancient recipe scented sachet (25); Decoction: Healthy Qi—invigorating Anti-Inflammatory Formula (35), Self-made Chinese medicinal formulae 1 (39), preventive prescription for H1N1 influenza (22); Tea substitute: Pihui Jiedu Prescription (26), Preventing influenza cold tea and disinfecting classrooms and dormitories with mugwort fumigation and steaming (37); Granule: Baopregnancy-kangdufang granules (23), Influenza Preventive Formulae (36), Fuzheng Gubiao granules (24); Chinese patent medicine: Qingjie Fanggan granules (27), Lianhua Qingwen capsule (38), antivirus granule (38); Drug combination: Lianhua Qingwen capsule plus antivirus granule (38)].

When we grouped the studies by intervention and compared TCM with blank controls, a total of 13 studies (22–29, 35–39) reported the incidence of influenza. Two of these studies were divided into three subgroups each: one study (38) reported results for three intervention groups, while another study (28) reported results for three time periods. Very low certainty of the evidence suggests that TCM may reduce the incidence of influenza (RR = 0.23, 95% CI 0.12–0.43, *P* <0.00001, *I*^2^ = 94%, 13 studies, 38,463 participants, Figure 4).

**FIGURE 5:**
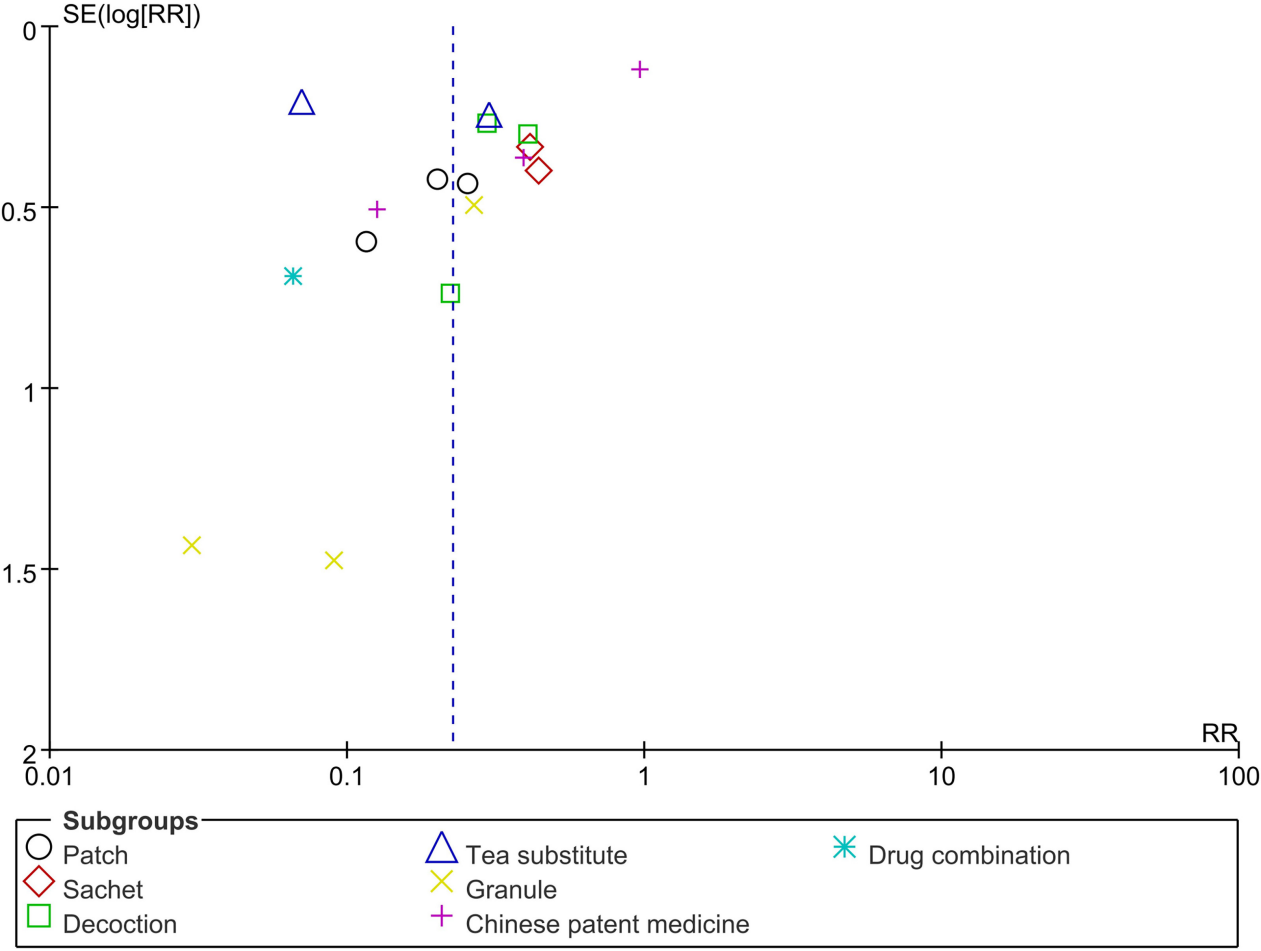
Funnel plot of influenza incidence.

In subgroup analyses by intervention type, Sanfu Tie (RR = 0.20, 95% CI 0.12–0.34, *P* < 0.00001, *I*^2^ = 0%, 1 study, 3 timepoints, 678 participants), sachets (RR = 0.43, 95% CI 0.26–0.70, *P* = 0.0009, *I*^2^ = 0%, 2 studies, 263 participants), decoctions (RR = 0.33, 95% CI 0.23–0.48, *P* < 0.00001, 3 study, 21,541 participants), herbal teas (RR = 0.15, 95% CI 0.03–0.65, *P* = 0.01, *I*^2^ = 95%, 2 studies, 4,650 participants), granules (RR = 0.14, 95% CI 0.03–0.59, *P* = 0.007, *I*^2^ = 33%, 3 studies, 11,012 participants), and combination therapies (RR = 0.07, 95% CI 0.02–0.26, *P* < 0.0001, 1 studies, 44 participants) may reduce the incidence of influenza. However, proprietary Chinese medicines did not show a statistically significant effect (RR = 0.38, 95% CI 0.07–2.04, *P* = 0.26, *I*^2^ = 95%, 3 studies, 275 participants, see Figure 4). Based on the observed asymmetry in the funnel plot, there may be potential publication bias in the included studies on this outcome measure (see Figure 5). The Egger’s test yielded a *P*-value of 0.021, indicating statistically significant small-study effects (i.e., publication bias) at the 5% significance level. The intercept of -3.024 demonstrated left-sided asymmetry in the funnel plot, suggesting that smaller studies showed more favorable preventive effects.

**FIGURE 6:**
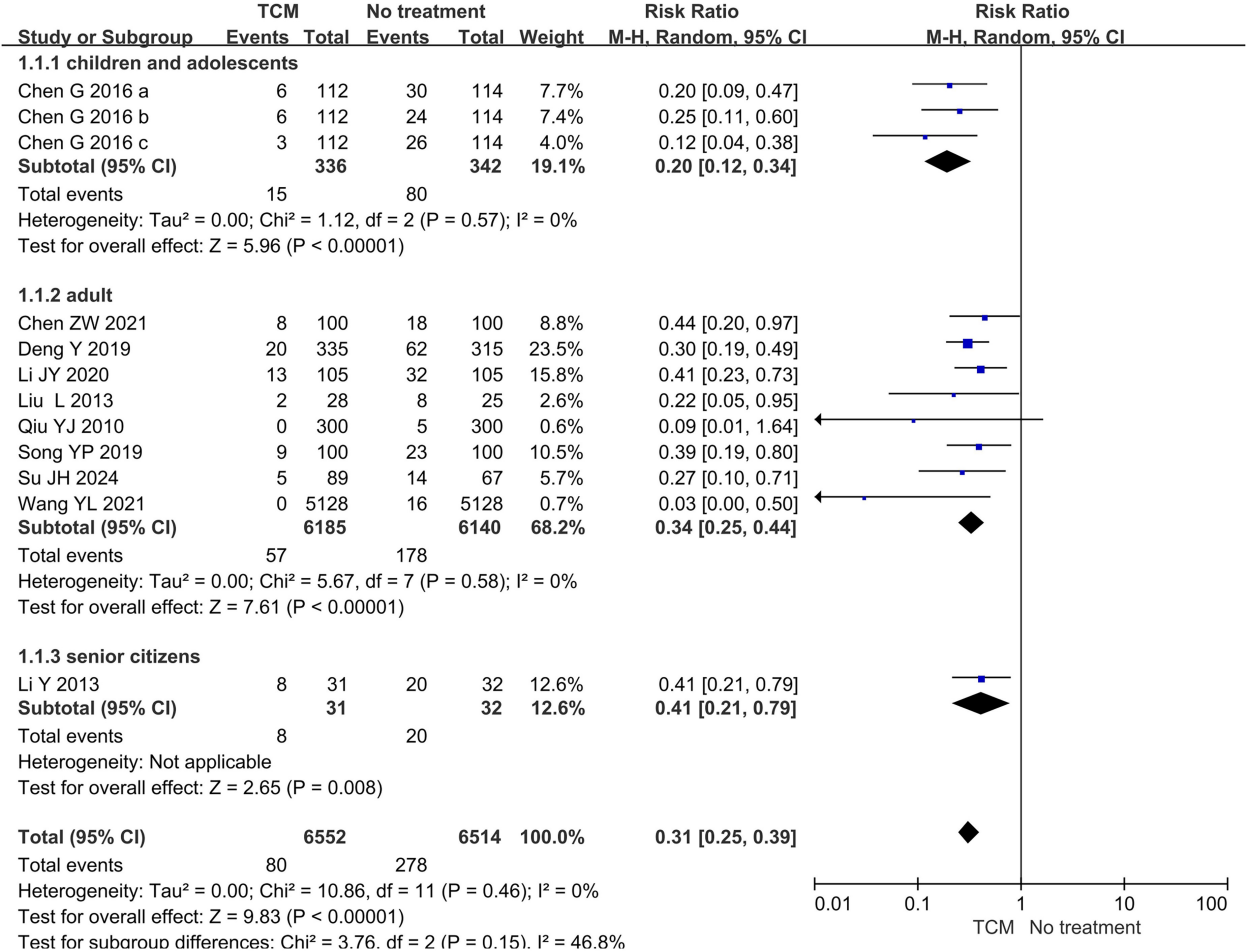
Forest plot of influenza incidence (subgroup by age).

In subgroup analyses by age, TCM preventive measures were associated with a lower incidence of influenza compared to no treatment across all age strata (children and adolescents: RR = 0.20, 95% CI 0.12–0.34, *P* < 0.00001, *I*^2^ = 0%, 1 study, 3 timepoints, 678 participants; adult: RR = 0.34, 95% CI 0.25–0.44, *P* < 0.00001, *I*^2^ = 0%, 8 studies, 12,325 participants; senior citizens: RR = 0.41, 95% CI 0.21–0.79, *P* = 0.008, 1 study, 63 participants). However, among the eight studies in the adult group, one study involving 600 participants (23) found no statistically significant difference between the TCM preventive measures and no treatment group (see Figure 6).

One cohort study (40) indicated that patients receiving TCM preventive measures may result in a reduction in the incidence of influenza when compared with blank controls (RR = 0.20, 95% CI 0.04–0.91, *P* = 0.04, 1 study, 768 participants; see Supplementary material 4).

#### 3.4.2 The incidence of influenza-like illness

Six studies (23, 26, 31–34) reported this outcome measure, all comparing TCM with blank controls. Very low certainty of the evidence suggests that TCM may reduce the incidence of influenza-like illness (RR = 0.38, 95% CI 0.23–0.63, *P* = 0.0002, *I*^2^ = 89%, 6 studies, 10,754 participants; Table 2 and Supplementary material 4).

**TABLE 2:** Outcome indicators evaluated Grade table.

Patient or population: healthy participants who do not have influenza may have any chronic disease. No restrictions on the participants’ gender, age, race or medical conditions.					
Setting: In hospital (9 studies), in school (6 studies), in community (2 studies), in the amred forces(1 study).					
Intervention: Traditional Chinese medicine (5 granules studies, 3 sachets studies, 5 Chinese herbal decoction studies, 2 tea substitute studies, 1 drug combination study, 1 Sanfu patch study, 1 Western medicine tablet study) Comparison: No treatment (17 studies), Indometacin (1 study)					
**Outcome No. of participants (studies)**	**Relative effect (95% CI)**	**Anticipated absolute effects (95% CI)**		**Difference**	**Certainty**
		**No treatment**	**Traditional Chinese medicine**		
Incidence of influenza No of participants: 38463 (17 RCTs)	RR 0.23 (0.12–0.43)	7.1%	1.6% (0.9–3.1)	5.5% fewer (6.3 fewer to 4.1 fewer)	⊕○○○ Very lowa,b
Incidence of influenza-like illness No of participants: 10754 (6 RCTs)	RR 0.38 (0.23–0.63)	9.7%	3.7% (2.2–6.1)	6.0% fewer (7.5 fewer to 3.6 fewer)	⊕○○○ Very lowa,b
Adverse reactions No of participants: 7486 (6 RCTs)	RR 5.00 (0.59–42.37)	0.0%	0.0% (0–0)	0.0% fewer (0 fewer to 0 fewer)	⊕○○○ Very lowa,c
Hospitalization rate No of participants: 650 (1 RCT)	Not estimable	0.0%	0.0% (0–0)	0.0% fewer (0 fewer to 0 fewer)	⊕○○○ Very lowa,c
Emergency (outpatient) attendance rate No. of participants: 650 (1 RCT)	RR 0.29 (0.19–0.47)	0.0%	0.0% (0–0)	0.0% fewer (0 fewer to 0 fewer)	⊕○○○ Very lowa,c
The risk in the intervention group (and its 95% confidence interval) is based on the assumed risk in the comparison group and the relative effect of the intervention (and its 95% CI). CI: confidence interval; RR: risk ratio					
GRADE Working Group grades of evidence					
High certainty: we are very confident that the true effect lies close to that of the estimate of the effect. Moderate certainty: we are moderately confident in the effect estimate: the true effect is likely to be close to the estimate of the effect, but there is a possibility that it is substantially different.					
Low certainty: our confidence in the effect estimate is limited: the true effect may be substantially different from the estimate of the effect.					
Very low certainty: we have very little confidence in the effect estimate: the true effect is likely to be substantially different from the estimate of effect.					

a. Downgraded by two levels because of within-study risk of bias. b. Downgraded by two levels because of within-study inconsistency. c. Downgraded by two levels because of within-study imprecision.

In subgroup analyses by intervention type, herbal teas (RR = 0.25, 95% CI 0.16–0.37, *P* < 0.00001, 1 study, 650 participants), sachets (RR = 0.31, 95% CI 0.12–0.81, *P* = 0.02, 1 study, 160 participants), and decoctions (RR = 0.40, 95% CI 0.30–0.53, *P* < 0.00001, *I*^2^ = 0%, 2 studies, 6,266 participants) may reduce the incidence of influenza-like illness, while granules showed no statistically significant effect (RR = 0.48, 95% CI 0.16–1.47, *P* = 0.2, *I*^2^ = 93%, 2 studies, 3,678 participants).

In subgroup analyses by age, adolescents (RR = 0.40, 95% CI 0.30–0.53, *P* < 0.00001, 2 studies, 6,266 participants) and adults (RR = 0.41, 95% CI 0.20–0.83, *P* = 0.01, 3 studies, 2,447 participants) receiving TCM preventive measures showed lower incidence rates of influenza-like illness compared to the blank control. However, no statistically significant difference was observed between the TCM preventive measures and no treatment group for senior citizens (RR = 0.96, 95% CI 0.73–1.26, *P* = 0.78, 3 studies, 1,391 participants; Supplementary material 4).

#### 3.4.3 Symptoms of influenza-like illness (TCM versus no treatment)

When compared with blank controls, TCM preventive measures may result in little to no difference in reducing the incidence of symptoms related to influenza-like illness (29, 31) (fever: RR = 0.84, 95% CI 0.05–13.78, *P* = 0.90, *I*^2^ = 68%, 2 studies, 360 participants; sore throat: RR = 0.84, 95% CI 0.05–13.78, *P* = 0.90, *I*^2^ = 68%, 2 studies, 360 participants; cough: RR = 0.67, 95% CI 0.12–3.81, *P* = 0.65, *I*^2^ = 19%, 2 studies, 360 participants; nasal congestion: RR = 2.00, 95% CI 0.18–21.71, *P* = 0.57, 1 study, 200 participants; runny nose: RR = 1.00, 95% CI 0.06–15.77, *P* = 1.00, 1 study, 200 participants; fatigue: RR = 0.25, 95% CI 0.05–1.14, *P* = 0.07, 1 study, 160 participants; see Supplementary material 4).

#### 3.4.4 Symptoms of influenza-like illness (TCM vs. Indometacin)

When compared with indomethacin, TCM preventive measures may result in a reduction in the incidence of symptoms related to influenza-like illness (30) (fever: RR = 0.49, 95% CI 0.26–0.94, *P* = 0.03, 1 study, 85 participants; fatigue: RR = 0.44, 95% CI 0.24–0.83, *P* = 0.01, 1 study, 85 participants; headache: RR = 0.34, 95% CI 0.13–0.87, *P* = 0.03, 1 study, 85 participants; muscle pain: RR = 0.42, 95% CI 0.22–0.82, *P* = 0.01, 1 study, 85 participants; anorexia: RR = 0.30, 95% CI 0.12–0.74, *P* = 0.009, 1 study, 85 participants; Supplementary material 4). However, no statistically significant difference was observed for chills (RR = 0.33, 95% CI 0.09–1.17, *P* = 0.09, 1 study, 85 participants; Supplementary material 4).

#### 3.4.5 Adverse events

**FIGURE 7:**
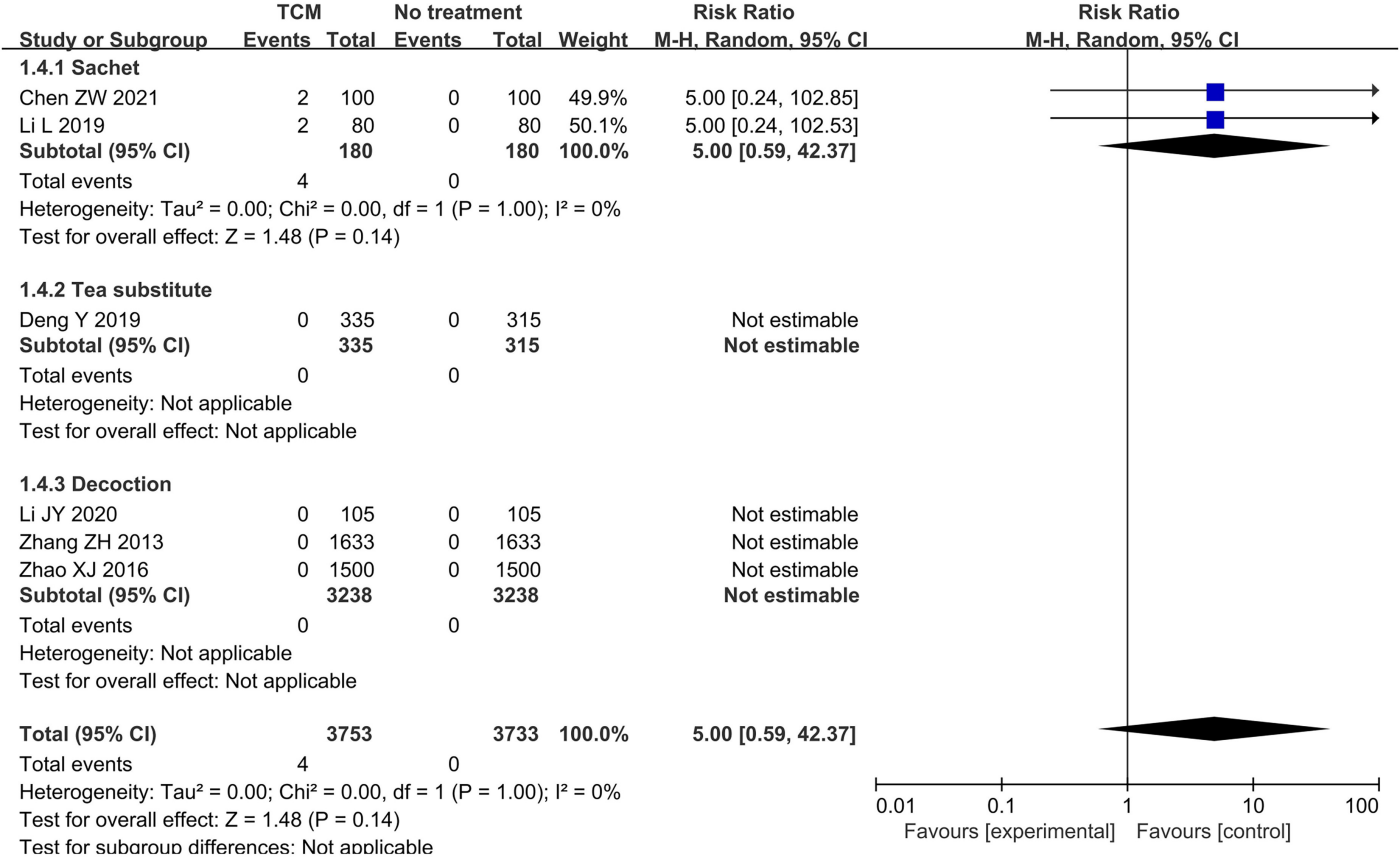
Forest plot of adverse reactions [Sachet: epidemic prevention sachets of traditional Chinese medicine (29), Influenza-Protective Herbal Sachet (5); Tea substitute: Pihui Jiedu Prescription (26); Decoction: Healthy Qi—invigorating Anti-Inflammatory Formula (35), Self-made Chinese medicinal formulae 2 (38), Self-made Chinese medicinal formulae 3 (32)].

The pooled analysis demonstrated no statistically significant difference in adverse events incidence between TCM interventions [involving sachets (29, 31), herbal teas (26), and herbal decoctions (32, 34, 35)] and no treatment (RR = 5.00, 95% CI 0.59–42.37, *P* = 0.14, *I*^2^ = 0%, 6 studies, 7,486 participants, Supplementary material 4), but the evidence was very uncertain. No adverse events were documented in either TCM intervention or blank control groups across the four studies involving herbal teas and herbal decoctions (Figure 7).

Analysis of the cohort study (40) showed that participants receiving Qinggandong Decoction may experience more adverse events when compared to the blank control (RR = 19.00, 95% CI 1.11–325.30, *P* = 0.04, 1 study, 768 participants, Supplementary material 4).

Subgroup analysis stratified by age demonstrated that neither the TCM preventive measures group nor the blank control group reported any adverse events in the two studies involving adolescents (32, 34). Furthermore, no statistically significant difference was observed in the incidence of adverse events between TCM prevention measures and blank controls among adult participants (RR = 5.00, 95% CI 0.59–42.37, *P* = 0.14, *I*^2^ = 0%, 6 studies, 7,486 participants, Supplementary material 4).

#### 3.4.6 Hospitalization rate

Very low certainty of the evidence indicated that Pihui Jiedu Prescription may reduce hospitalization rates or emergency (outpatient) visit rates (RR = 0.29, 95% CI 0.19–0.47, *P* < 0.00001, 1 study, 1,300 participants, Table 2 and Supplementary material 4) when compared with no treatment.

## 4 Discussion

This review identified 12,197 candidate studies, with 19 studies involving 55,183 healthy participants ultimately meeting inclusion criteria. This review comprised specific populations, including 600 pregnant women, 226 children, 31,889 school students, 3,238 community residents, and 210 military residents.

The direct comparison results indicate that TCM preventive measures may reduce the incidence of influenza and influenza-like illnesses, decrease hospitalization rates or emergency/outpatient visits, and may exhibit safety. Sanfu plaster, sachets, herbal decoctions, herbal teas, granules, and combination therapies may all contribute to reducing the incidence of influenza. Herbal teas, sachets, and decoctions may have a positive effect on reducing the incidence of influenza-like illnesses. Prophylactic use of indomethacin may reduce the occurrence of influenza-like symptoms such as fever, fatigue, headache, myalgia, and anorexia. The Pihui Jiedu Prescription may lower the hospitalization or emergency/outpatient visit rates due to influenza in healthy populations. Subgroup analyses revealed that TCM preventive measures may reduce the incidence of influenza in children and adolescents, adults, and the elderly. TCM preventive measures may result in a reduction in the incidence of influenza-like illness for adolescents and adults, while no significant benefit was observed in the elderly population. The application of TCM preventive measures appears to be safe in adolescents and adults. However, insufficient safety data currently exist regarding their application in elderly populations.

The inclusion of pregnant women, children, elderly with chronic bronchitis, and military personnel highlights the broad applicability of TCM across populations where conventional options may be limited. Pregnant women face uncertain vaccination risks, young children may resist standard interventions, and elderly individuals often show reduced vaccine response. The positive findings across these groups suggest TCM may fill important gaps in current prevention strategies. The particularly strong effect in pregnant women (5.0% vs. 20.3%) may reflect the individualized approach of Baopregnancy-kangdufang granules (23). Sanfu plaster in children combines acupoint stimulation with transdermal absorption, offering a suitable option for young children (28). Among elderly chronic bronchitis patients, scented sachets reduced both influenza incidence and illness duration (2.0 vs. 6.0 days) (25), suggesting benefits beyond infection prevention. Successful implementation in military personnel (35) demonstrates feasibility in congregate settings. These real-world applications, combined with favorable safety profiles, support TCM’s potential role within broader public health frameworks.

Although direct comparisons with conventional preventive measures were not available in the included studies, contextualization with existing evidence may provide perspective. As noted in the introduction, influenza vaccines offer average effectiveness of approximately 60% (7), while oseltamivir has demonstrated 74% preventive efficacy in healthy adults (8). An overview by our team previously suggested that TCM interventions such as Ganmao Capsules showed potential for influenza prevention in indirect comparisons (12). In the present analysis, TCM was associated with a risk ratio of 0.23 for influenza incidence. However, this estimate is derived from very low certainty evidence with high heterogeneity and should not be directly compared to vaccine or antiviral effectiveness. The apparent effect size may be influenced by methodological limitations of the included studies, as discussed above. Beyond China, other Asian countries also have traditions of using herbal medicines for influenza prevention. In Japan, a Kampo medicine called Hochuekkito (TJ-41) was studied in mice. Pretreatment with TJ-41 for 2 weeks before influenza virus infection reduced viral replication, accompanied by increased GM-CSF expression and elevated defensin mRNA levels (42). In India, a survey during the 2009 influenza pandemic found that herbal remedies were the most widely reported home treatment. Rural respondents emphasized climatic conditions, contaminated water, and humoral imbalance from hot or cold foods as perceived causes of influenza, reflecting traditional concepts (43). In Iran, a review of Iranian Traditional Medicine identified numerous medicinal plants for cold and flu prevention, including antiviral plants (honeysuckle, thyme, andrographis, peppermint), expectorant plants (tulsi, licorice root, clove, sage), and immunostimulant plants (eucalyptus, ginseng, garlic, ginger, Isatis root) (44). These diverse traditional medicine systems across Asia share common emphasis on herbal approaches for respiratory infection prevention.

Existing literature partially supports the potential effectiveness of TCM in influenza prevention. A mechanistic study of Lianhua Qingwen capsules (45) demonstrated four primary antiviral actions: (1) inhibition of viral attachment to host cells; (2) suppression of viral replication and release; (3) downregulation of chemokine/cytokine expression; (4) immune system modulation. These pharmacological properties suggest clinical utility for influenza treatment and prevention. The preventive effects observed in this review may be supported by pharmacological mechanisms reported in previous studies. Maxingshigan Decoction enhances IL-2 and IL-4 expression and regulates T-cell subsets (14, 15). Catechin extract inhibits influenza A virus replication (17), while mixed polysaccharides enhance resistance to H1N1 infection and reduce lung injury (18). These diverse mechanisms—including immunomodulation and direct antiviral activity—may collectively contribute to the preventive effects observed in our meta-analysis.

A Cochrane systematic review (46) that included 18 RCTs involving 2,521 participants found similar effectiveness between most Chinese herbal formulations and conventional antivirals. Few Chinese herbal medicines have been proven superior to antiviral drugs, and the current evidence remains limited in reliability and validity due to methodological constraints in the trials. These findings are consistent with our results. Similarly, both COVID-19 and influenza are viral infectious diseases caused by viruses, sharing similarities in transmission routes and clinical manifestations. A meta-analysis (47) has demonstrated the potential of TCM in treating COVID-19. The experience gained from using TCM to prevent COVID-19 infection may therefore also be applicable to influenza prevention.

### 4.1 Strengths and limitations

This review is the first comprehensive study to analyze the efficacy and safety of TCM interventions for influenza prevention using direct comparison methods, providing a relatively systematic and thorough summary of the evidence. This review also reveals that TCM measures have certain potential in preventing influenza. However, several limitations warrant consideration. At present, most of the research focuses on treating influenza, while research on preventing influenza is relatively scarce. Therefore, this analysis incorporated only 19 primary studies, constituting an inadequate sample size for robust conclusions. Substantial missing data further constrained our risk-of-bias assessment, contributing to the overall low methodological quality of included studies and consequently limiting confidence in the evidence certainty. Due to the limited number of included studies, the data on safety obtained is very limited, which also makes it impossible for us to conduct a detailed analysis of the evidence on safety. While we conducted subgroup analyses to address variability, significant heterogeneity persisted in quantitative syntheses due to variations in study designs and baseline population characteristics, necessitating careful interpretation of these results. Moreover, substantial heterogeneity existed in the diagnostic methods for influenza and influenza-like illness across included studies, ranging from laboratory-confirmed testing to clinical definitions alone. This variability in case ascertainment may introduce misclassification bias and further contribute to the high statistical heterogeneity observed in our pooled analyses. In addition, due to inconsistent reporting of follow-up time points across the included studies, we were unable to perform subgroup analyses based on different time intervals after intervention. This precludes any assessment of how the preventive effects of TCM may vary over time, such as during the immediate post-intervention period versus longer-term follow-up. The lack of time-stratified analysis limits our understanding of the durability of any preventive effect and may obscure important temporal patterns in influenza incidence. Additionally, funnel plot analyses and egger’s test indicated potential publication bias for certain outcomes. Furthermore, the included studies exhibited substantial variation in sample size (ranging from 44 to over 30,000 participants). Although we used random-effects models to mitigate the disproportionate influence of large studies, the possibility remains that results may be disproportionately influenced by a few large trials. Future updates of this review should consider individual patient data meta-analysis or more sophisticated sensitivity analyses when more studies become available. In summary, the current meta-analysis provides insufficient confidence to support definitive conclusions.

### 4.2 Implications for future research

The reviewed studies exhibited considerable inconsistency in defining influenza, with many failing to distinguish it clearly from common colds, upper respiratory tract infections, and other clinically similar conditions. During the search process, we found that the proportion of studies related to “treating influenza” was higher, while the number of studies on “preventing influenza” was very limited. Therefore, we hope that in the future, more research will focus on “TCM for preventing influenza.” Additionally, there is a scarcity of studies with high methodological quality. We believe that rigorous methods and high-quality study designs are essential to improve the validity of future evidence. In particular, future studies should adopt standardized, laboratory-confirmed diagnostic methods for influenza, with clear reporting of case definitions and testing procedures. For influenza-like illness, researchers should explicitly cite and adhere to established guidelines to ensure consistency across studies. Future studies should also report influenza incidence at multiple standardized time points after intervention, such as at 1, 3, and 6 months, to enable time-stratified meta-analyses. This would allow researchers to assess the durability of preventive effects and identify the optimal duration of protection conferred by TCM interventions. Consistent reporting of follow-up schedules across studies is essential for such analyses. Furthermore, attention should be given to outcome measures such as the incidence of influenza and influenza-like illnesses, adverse event frequencies, and healthcare utilization metrics (e.g., outpatient visits and hospitalizations), as these are critical for evaluating the role of TCM in influenza prevention. Future investigations should prioritize precise case definitions, rigorous randomization techniques (e.g., random number table method, block random method) and allocation concealment (e.g., sealed opaque envelopes method) methods, addressing missing data appropriately, identifying core outcome measures for influenza prevention, and ensuring comprehensive and systematic reporting of results. Furthermore, researchers should strive to make the sample sizes of each research group more balanced, in order to reduce the risk that individual large-scale studies in future meta-analyses may have a dominant influence on the overall analysis.

## 5 Conclusion

TCM demonstrates potential efficacy in preventing influenza. However, due to the limitations of clinical studies related to influenza prevention in healthy populations, particularly their low methodological quality, which constrains our confidence in these findings. Very low certainty of evidence suggests that TCM may have a potential role in reducing the incidence of influenza and influenza-like illnesses while maintaining a potential safety profile. Notably, we found no high-certainty evidence supporting the efficacy or safety of any preventive measures examined. To strengthen future research, we emphasize the need for precise influenza case definitions, improved study design methodologies, and implementation of standardized outcome measures specifically tailored for influenza prevention studies.

## Data Availability

The original contributions presented in the study are included in the article/Supplementary material, further inquiries can be directed to the corresponding author.
