# Supplementary material for: Traditional Chinese Medicine for preventing influenza: a systematic review and meta-analysis
Source: Front Med (Lausanne). 2026 Apr 23;13:1736574. doi: 10.3389/fmed.2026.1736574 (PMC13149241; doi:10.3389/fmed.2026.1736574)
Supplement: Supplementary file 1 [file Data_Sheet_1.pdf]

Supplementary materials 1 The retrieval strategy

Archive	Search mode
CNKI (Chinese database)	(SU%='流行性感冒' OR SU%='流感' OR SU%='人类流感' OR SU%='人流感' OR SU%='甲型流感' OR SU%='甲流' OR SU%='乙型流感' OR SU%='乙流' OR SU%='感冒') AND (SU%='中医药' OR SU%='中医' OR SU%='传统医学' OR SU%='中药汤剂' OR SU%='中成药' OR SU%='中药香囊' OR SU%='中药贴敷' OR SU%='针刺' OR SU%='艾灸' OR SU%='针灸' OR SU%='拔火罐' OR SU%='拔罐' OR SU%='推拿') AND (SU%='临床研究' OR SU%='随机对照试验' OR SU%='随机' OR SU%='对照' OR SU%='临床对照试验' OR SU%='对照试验' OR SU%='队列研究' OR SU%='队列' OR SU%='暴露' OR SU%='分组')
Wang Fang (Chinese database)	主题: (“流行性感冒"or“流感"or“人类流感"or“人流感"or“甲型流感"or“甲流"or“乙型流感"or“乙流"or“感冒")AND 主题:(“中医药"or“中医"or“传统医学"or“中药汤剂"or“中成药"or“中药香囊"or“中药贴敷"or“针刺"or“艾灸"or“针灸"or“拔火罐"or“拔罐"or“推拿")AND 主题:(“临床研究"or“随机对照试验"or“随机"or“对照"or“临床对照试验"or“对照试验"or“队列研究"or“队列"or“暴露"or“分组")
Sinomed (Chinese database)	(("暴露"[常用字段:智能] OR "分组"[常用字段:智能]) OR ("临床研究"[常用字段:智能] OR "随机对照试验"[常用字段:智能] OR "随机"[常用字段:智能] OR "对照"[常用字段:智能] OR "临床对照试验"[常用字段:智能] OR "对照试验"[常用字段:智能] OR "队列研究"[常用字段:智能] OR "队列"[常用字段:智能])) AND (("针灸"[常用字段:智能] OR "拔火罐"[常用字段:智能] OR "拔罐"[常用字段:智能] OR "推拿"[常用字段:智能]) OR ("中医药"[常用字段:智能] OR "中医"[常用字段:智能] OR "传统医学"[常用字段:智能] OR "中药汤剂"[常用字段:智能] OR "中成药"[常用字段:智能] OR "中药香囊"[常用字段:智能] OR "中药贴敷"[常用字段:智能] OR "针刺"[常用字段:智能] OR "艾灸"[常用字段:智能])) AND ("流行性感冒"[常用字段:智能] OR "流感"[常用字段:智能] OR "人类流感"[常用字段:智能] OR "人流感"[常用字段:智能] OR "甲型流感"[常用字段:智能] OR "甲流"[常用字段:智能] OR "乙型流感"[常用字段:智能] OR "乙流"[常用字段] OR "感冒"[常用字段:智能])
CQVIP (Chinese database)	(U=流行性感冒 OR U=流感 OR U=人类流感 OR U=人流感 OR U=甲型流感 OR U=甲流 OR U=乙型流感 OR U=乙流 OR U=感冒) AND (U=中医药 OR U=中医 OR U=传统医学 OR U=中药汤剂 OR U=中成药 OR U=中药香囊 OR U=中药贴敷 OR U=针刺 OR U=艾灸 OR U=针灸 OR U=拔火罐 OR U=拔罐 OR U=推拿) AND (U=临床研究 OR U=随机对照试验 OR U=随机 OR U=对照 OR U=临床对照试验 OR U=对照试验 OR U=队列研究 OR U=队列 OR U=暴露 OR U=分组)

PubMed	#1 Search: ((((((((((((((((((((((Traditional Chinese Medicine[MeSH Terms]) OR (Traditional Chinese Medicine[Title/Abstract])) OR (Chung I Hsueh[Title/Abstract])) OR (Hsueh, Chung I[Title/Abstract])) OR (Traditional Medicine, Chinese[Title/Abstract])) OR (Zhong Yi Xue[Title/Abstract])) OR (Chinese Traditional Medicine[Title/Abstract])) OR (Chinese Medicine, Traditional[Title/Abstract])) OR (Traditional Tongue Diagnosis[Title/Abstract])) OR (Tongue Diagnoses, Traditional[Title/Abstract])) OR (Tongue Diagnosis, Traditional[Title/Abstract])) OR (Traditional Tongue Diagnoses[Title/Abstract])) OR (Traditional Tongue Assessment[Title/Abstract])) OR (Tongue Assessment, Traditional[Title/Abstract])) OR (Traditional Tongue Assessments[Title/Abstract])) OR (Chinese medicine decoction[Title/Abstract])) OR (Chinese patent medicine[Title/Abstract])) OR (Chinese medicine sachets[Title/Abstract])) OR (Chinese medicine patch[Title/Abstract])) OR (Acupuncture[Title/Abstract])) OR (Moxibustion[Title/Abstract])) OR (Cupping[Title/Abstract])) OR (Massage[Title/Abstract])<br><br>#2 Search: ((((((((((Influenza, Human[MeSH Terms]) OR (Human Influenzas[Title/Abstract])) OR (Influenzas, Human[Title/Abstract])) OR (Influenza[Title/Abstract])) OR (Influenzas[Title/Abstract])) OR (Human Flu[Title/Abstract])) OR (Flu, Human[Title/Abstract])) OR (Human Influenza[Title/Abstract])) OR (Influenza in Humans[Title/Abstract])) OR (Influenza in Human[Title/Abstract])) OR (Grippe[Title/Abstract])<br><br>#3 Search: (#1) AND (#2)
EMbase	#1: 'traditional chinese medicine'/exp OR 'traditional chinese medicine':ti,ab,kw OR 'chung i hsueh':ti,ab,kw OR 'hsueh, chung i':ti,ab,kw OR 'traditional medicine, chinese':ti,ab,kw OR 'zhong yi xue':ti,ab,kw OR 'chinese traditional medicine':ti,ab,kw OR 'chinese medicine, traditional':ti,ab,kw OR 'traditional tongue diagnosis':ti,ab,kw OR 'tongue diagnoses, traditional':ti,ab,kw OR 'tongue diagnosis, traditional':ti,ab,kw OR 'traditional tongue diagnoses':ti,ab,kw OR 'traditional tongue assessment':ti,ab,kw OR 'tongue assessment, traditional':ti,ab,kw OR 'traditional tongue assessments':ti,ab,kw OR 'chinese medicine decoction':ti,ab,kw OR 'chinese patent medicine':ti,ab,kw OR 'chinese medicine sachets':ti,ab,kw OR 'chinese medicine patch':ti,ab,kw OR acupuncture:ti,ab,kw OR moxibustion:ti,ab,kw OR

	cupping:ti,ab,kw OR massage:ti,ab,kw<br><br>#2: 'influenza, human'/exp OR 'human influenzas':ti,ab,kw OR 'influenzas, human':ti,ab,kw OR influenza:ti,ab,kw OR influenzas:ti,ab,kw OR 'human flu':ti,ab,kw OR 'flu, human':ti,ab,kw OR 'human influenza':ti,ab,kw OR 'influenza in humans':ti,ab,kw OR 'influenza in human':ti,ab,kw OR grippe:ti,ab,kw<br><br>#3：#1 AND #2
CENTRAL	#1：(Traditional Chinese Medicine):ti,ab,kw OR (Chung I Hsueh):ti,ab,kw OR (Hsueh, Chung I):ti,ab,kw OR (Traditional Medicine, Chinese):ti,ab,kw OR (Zhong Yi Xue):ti,ab,kw<br>#2：(Chinese Traditional Medicine):ti,ab,kw OR (Chinese Medicine, Traditional):ti,ab,kw OR (Traditional Tongue Diagnosis):ti,ab,kw OR (Tongue Diagnoses, Traditional):ti,ab,kw OR (Tongue Diagnosis, Traditional):ti,ab,kw<br>#3：(Traditional Tongue Diagnoses):ti,ab,kw OR (Traditional Tongue Assessment):ti,ab,kw OR (Tongue Assessment, Traditional):ti,ab,kw OR (Traditional Tongue Assessments):ti,ab,kw OR (Chinese medicine decoction):ti,ab,kw<br>#4：(Chinese patent medicine):ti,ab,kw OR (Chinese medicine sachets):ti,ab,kw OR (Chinese medicine patch):ti,ab,kw OR (Acupuncture):ti,ab,kw OR (Moxibustion):ti,ab,kw<br>#5：(Cupping):ti,ab,kw OR (Massage):ti,ab,kw<br>#6：MeSH descriptor: [Medicine, Chinese Traditional] explode all trees<br>#7：#1 OR #2 OR #3 OR #4 OR #5 OR #6<br>#8：(Human Influenzas):ti,ab,kw OR (Influenzas, Human):ti,ab,kw OR (Influenza):ti,ab,kw OR (Influenzas):ti,ab,kw OR (Human Flu):ti,ab,kw<br>#9：(Flu, Human):ti,ab,kw OR (Human Influenza):ti,ab,kw OR (Influenza in Humans):ti,ab,kw OR (Influenza in Human):ti,ab,kw OR (Grippe):ti,ab,kw<br>#10：MeSH descriptor: [Influenza, Human] explode all trees<br>#11：#8 OR #9 OR #10<br>#12：#7 AND #11
Web of Science	Query #1：((((((((((((((((((((TS=(Traditional Chinese Medicine)) OR TS=(Chung I Hsueh)) OR TS=(Hsueh, Chung I))

	OR TS=(Traditional Medicine, Chinese)) OR TS=(Zhong Yi Xue)) OR TS=(Chinese Traditional Medicine)) OR TS=(Chinese Medicine, Traditional)) OR TS=(Traditional Tongue Diagnosis)) OR TS=(Tongue Diagnoses, Traditional)) OR TS=(Tongue Diagnosis, Traditional)) OR TS=(Traditional Tongue Diagnoses)) OR TS=(Traditional Tongue Assessment)) OR TS=(Tongue Assessment, Traditional)) OR TS=(Traditional Tongue Assessments)) OR TS=(Chinese medicine decoction)) OR TS=(Chinese patent medicine)) OR TS=(Chinese medicine sachets)) OR TS=(Chinese medicine patch)) OR TS=(Acupuncture)) OR TS=(Moxibustion)) OR TS=(Cupping)) OR TS=(Massage)<br><br>Query #2： ((((((((((TS=(Influenza, Human)) OR TS=(Human Influenzas)) OR TS=(Influenzas, Human)) OR TS=(Influenza)) OR TS=(Influenzas)) OR TS=(Human Flu)) OR TS=(Flu, Human)) OR TS=(Human Influenza)) OR TS=(Influenza in Humans)) OR TS=(Influenza in Human)) OR TS=(Grippe)<br><br>#3：#1 AND #2
