# Supplementary material for: Traditional Chinese Medicine for preventing influenza: a systematic review and meta-analysis
Source: Front Med (Lausanne). 2026 Apr 23;13:1736574. doi: 10.3389/fmed.2026.1736574 (PMC13149241; doi:10.3389/fmed.2026.1736574)
Supplement: Supplementary file 2 [file Data_Sheet_2.pdf]

Supplementary Materials 2 The preparation and composition of the Chinese herbal medicines in the included trials

Name of herbs	Preparation	Composition (English name, Latin name)	Study ID
preventive prescription for H1N1 influenza	Decoction	Zi Cao (Gromwell Root, Arnebiae Radix), Bo He (Mint, Menthae Haplocalycis Herba), Gan Cao (Liquorice root, Glycyrrhizae Radix Et Rhizoma)	Liu L 2013
Preventing influenza cold tea and disinfecting classrooms and dormitories with mugwort fumigation and steaming	Decoction	Jin Yin Hua(Honeysuckle Flower, Lonicerae Japonicae Flos),Ban Lan Gen(Indigowoad Root, Isatidis Radix),Zhu Ye(Common Lophatherum Herb, Lophatheri Herba),Ge Gen(Lobed Kudzuvine Root, Puerariae Lobatae Radix),Niu Bang Zi(Great Burdock Fruit, Arctii Fructus),Huo Xiang(Cablin Potchouli Herb, Pogostemonis Herba),Jie Geng(Platycodon Grandiflorum, Platycodonis Radix), Su Ye(Cultibated Purple Perilla Leaf, Perillae Folium),Bai Mao Gen(Lalang grass rhizome, Imperatae Rhizoma),Gan Cao(Liquorice root, Glycyrrhizae Radix Et Rhizoma)	Luo Y 2013
Baopregnancy-kangdufang granule	Granule	Bai Zhu (Largehead Atractylodes Rh, Atractylodis Macrocephalae Rhizoma), Huang Qin (Baical skullcap root, Scutellariae Radix), Ban Lan Gen (Indigowoad Root, Isatidis Radix), Tai Zi Shen (Heterophylly Falsestarwort Root, Pseudostellariae Radix), Su Ye (Cultibated Purple Perilla Leaf, Perillae Folium), Fang Feng (Divaricate Saposhniovia Root, Saposhnikoviae Radix), Gan Cao (Liquorice root, Glycyrrhizae Radix Et Rhizoma)	Qiu YJ 2010
Fuzheng Gubiao granules	Granule	Huang Qi(Milkvetch Root, Astragali Radix),Chai Hu(Chinese Thorowax Root, Bupleuri Radix),Fa Ban	Wang YL 2021

		Xia(Pinellia Tuber, Pinelliae Rhizoma Praeparatum),Dang Shen(Tangshen, Codonopsis Radix),Da Zao(Fructus Jujubae, Jujubae Fructus), Bai Zhu (Largehead Atractylodes Rh, Atractylodis Macrocephalae Rhizoma), Fang Feng(Divaricate Saposhniovia Root, Saposhnikoviae Radix),Huo Xiang(Cablin Potchouli Herb, Pogostemonis Herba),Pei Lan(Eupatorium fortunei Turcz, Eupatorii Herba),Lian Qiao(Weeping Forsythia Capsule, Forsythiae Fructus),Huang Qin(Baical skullcap root, Scutellariae Radix) ,Zhi Gan Cao(Honey-fried licorice root, Glycyrrhizae Radix Et Rhizoma Praeparata Cum Melle)。	
Ancient recipe scented sachet	Powder	Qiang Huo (Incised notopterygium rhizome and root, Notopterygii Rhizoma Et Radix), Da Huang (Rhubarb root and rhizome, Rhei Radix Et Rhizoma), Chai Hu (Chinese Thorowax Root, Bupleuri Radix), Cang Zhu (Rhizoma Atractylodis, Atractylodis Rhizoma), Xi Xin (Manchurian wildginger, Asari Radix Et Rhizoma), Wu Zhu Yu (Medcinal Evodia Fruit, Euodiae Fructus), Ding Xiang (Clove, Caryophylli Flos), Bing Pian (Synthetic Borneol, Borneolum Syntheticum)	Li Y 2013
Cure foul detoxification prescription for tea drink	Tea substitute	Cang Zhu (Rhizoma Atractylodis, Atractylodis Rhizoma), Chen Pi (Tangerine Peel, Citri Reticulatae Pericarpium), Jin Yin Hua (Honeysuckle Flower, Lonicerae Japonicae Flos), Bai Mao Gen (Lalang grass rhizome, Imperatae Rhizoma), Sang Ye (Mulberry Leave, Mori Folium)	Deng Y 2019

Qingjie Fanggan granules	Granule	Jin Yin Hua (Honeysuckle Flower, Lonicerae Japonicae Flos), Xuan Shen (Figwort Root, Scrophulariae Radix), Chen Pi (Tangerine Peel, Citri Reticulatae Pericarpium), Gan Cao (Liquorice root, Glycyrrhizae Radix Et Rhizoma), Da Zao (Fructus Jujubae, Jujubae Fructus),	Song YP 2019
Sanfu plaster	Powder	Bai Jie Zi (White Mustard Seed, Sinapis alba L), Xi Xin (Manchurian wildginger, Asari Radix Et Rhizoma), Yan Hu Suo (Corydalis Yanhusuo, Corydalis Rhizoma), Gan Sui (Kansui Root, Kansui Radix), Sheng Jiang (Ginger, Zingiberis Rhizoma Recens)	Chen G 2016
epidemic prevention sachets of traditional Chinese medicine	Powder	Cang Zhu (Rhizoma Atractylodis, Atractylodis Rhizoma), Chuan Xiong (Sichuan lovase rhizome, Chuanxiong Rhizoma), Bai Zhi (Angelica dahurica, Angelicae Dahuricae Radix), Ai Ye (Chinese mugwort, Artemisiae Argyi Folium), Guang Huo Xiang (Cablin Potchouli Herb, Pogostemonis Herba), Pei Lan (Eupatorium fortunei Turcz, Eupatorii Herba), Bo He (Mint, Menthae Haplocalycis Herba),	Chen ZW 2021
Xinhuang Pian	Pill	Zhong Jie Feng (Glabrous Sarcandra Herb, Sarcandrae Herba), San Qi (Sanchi, Notoginseng Radix Et Rhizoma), Ren Gong Niu Huang (Calculus bovis factitius, Bovis Calculus Artifactus), Xiao Fan Tian Hua (Herb of Procumbent Indian Mallow, Urena lobata Linn), Zhen Zhu (Pearl, Margarita), Shui Niu Jiao Nong Suo Fen (Cornu Bubali, Pulvis Cornus Bubali Concentratus), Hong Qu (Red rice, Fermentum rubrum)	Wang ZW 2008
①Lianhua Qingwen capsule ② Lianhua Qingwen capsule +	Capsule/ granule	Lianhua Qingwen capsule： Lian Qiao(Weeping Forsythia Capsule, Forsythiae Fructus),Jin Yin	Zhang M 2010

antivirus granule；③ antivirus granule		Hua(Honeysuckle Flower, Lonicerae Japonicae Flos),Ma Huang(Chinese Ephedrs Herb, Ephedrae Herba),Ku Xin Ren(Bitter Apricot Seed, Armeniacae Semen Amarum),Shi Gao(Gypsum, Gypsum Fibrosum),Ban Lan Gen(Indigowoad Root, Isatidis Radix),Mian Ma Guan Zhong(Rhizoma dryopteris crassirhizomae, Dryopteridis Crassirhizomatis Rhizoma),Yu Xing Cao(Heartleaf Houttuynia Herb, Houttuyniae Herba),Guang Huo Xiang(Cablin Potchouli Herb, Pogostemonis Herba),Da Huang(Rhubarb root and rhizome, Rhei Radix Et Rhizoma),Hong Jing Tian(Rhodiola rosea, Rhodiolae Crenulatae Radix Et Rhizoma),Bo He Nao(Menthol, l-Menthol),Gan Cao(Liquorice root, Glycyrrhizae Radix Et Rhizoma)<br>antivirus granule： Ban Lan Gen(Indigowoad Root, Isatidis Radix),Ren Dong Teng(Caulis Lonicerae, Lonicerae Japonicae Caulis),Shan Dou Gen(Vietnamese Sophora Root, Sophorae Tonkinensis Radix Et Rhizoma),Chuan She Gan(Blackberry lily, Iridis Tectori Rhizoma),Yu Xing Cao(Heartleaf Houttuynia Herb, Houttuyniae Herba),Chong Lou(Yunnan Manyleaf Paris Rhizome, Paridis Rhizoma),Guan Zhong(Japanese Flowering Fern Rhizome, Dryopteridis Crassirhizomatis Rhizoma),Bai Zhi (Angelica dahurica, Angelicae Dahuricae Radix),Qing Hao (Sweet Wormwood Herb, Artemisiae Annuae Herba)	
Traditional Chinese	Powder	Guang Huo Xiang (Cablin Potchouli	Li L 2019

medicine anti-influenza sachets		Herb, Pogostemonis Herba), Cang Zhu (Rhizoma Atractylodis, Atractylodis Rhizoma), Shi Chang Pu (Acorus Tatarinowii, Acori Tatarinowii Rhizoma), Ding Xiang (Clove,Caryophylli Flos)	
Self-made Chinese medicinal formulae 3	Decoction	Zhu Ye(Common Lophatherum Herb, Lophatheri Herba), Gan Cao(Liquorice root, Glycyrrhizae Radix Et Rhizoma), Ban Lan Gen(Indigowoad Root, Isatidis Radix), Yu Xing Cao(Heartleaf Houttuynia Herb, Houttuyniae Herba),Shan Zha(Crataegus Pinnatifida, Crataegi Fructus), Shi Gao(Gypsum, Gypsum Fibrosum), Huang Qin(Baical skullcap root, Scutellariae Radix) ,Niu Bang Zi(Great Burdock Fruit, Arctii Fructus), Xin Yi(Biod Magnolia Bud, Magnoliae Flos), Lian Qiao(Weeping Forsythia Capsule, Forsythiae Fructus), Jin Yin Hua(Honeysuckle Flower, Lonicerae Japonicae Flos), Da Qing Ye(Indigowoad Leaf, Isatidis Folium),Guan Zhong(Japanese Flowering Fern Rhizome, Dryopteridis Crassirhizomatis Rhizoma)	Zhao XJ 2016
preventive decoction of adults or the elderly	Granule	Preventive decoction of adults： Jin Yin Hua(Honeysuckle Flower, Lonicerae Japonicae Flos) ,Da Qing Ye(Indigowoad Leaf, Isatidis Folium),Bo He(Mint, Menthae Haplocalycis Herba),Gan Cao(Liquorice root, Glycyrrhizae Radix Et Rhizoma)<br>Preventive decoction of the elderly：<br>Tai Zi Shen(Heterophylly Falsestarwort Root, Pseudostellariae Radix) ,Su Ye(Cultibated Purple	Su HP 2010

Self-made Chinese medicinal formulae 2	Decoction	Perilla Leaf, Perillae Folium),Huang Qin(Baical skullcap root, Scutellariae Radix) ,Niu Bang Zi (Great Burdock Fruit, Arctii Fructus) Guan Zhong(Japanese Flowering Fern Rhizome, Dryopteridis Crassirhizomatis Rhizoma),Da Qing Ye(Indigowoad Leaf, Isatidis Folium),Jin Yin Hua(Honeysuckle Flower, Lonicerae Japonicae Flos),Lian Qiao(Weeping Forsythia Capsule, Forsythiae Fructus),Xin Yi(Biod Magnolia Bud, Magnoliae Flos),Niu Bang Zi(Great Burdock Fruit, Arctii Fructus),Huang Qin(Baical skullcap root, Scutellariae Radix) ,Shi Gao(Gypsum, Gypsum Fibrosum),Shan Zha (Crataegus Pinnatifida, Crataegi Fructus),Yu Xing Cao(Heartleaf Houttuynia Herb, Houttuyniae Herba),Ban Lan Gen(Indigowoad Root, Isatidis Radix),Gan Cao(Liquorice root, Glycyrrhizae Radix Et Rhizoma), Zhu Ye(Common Lophatherum Herb, Lophatheri Herba)。	Zhang 2013	ZH
Qinggandong Decoction	Tea substitute	Huang Qi(Milkvetch Root, Astragali Radix), Hu Zhang(Giant Knotweed Rhizome, Polygoni Cuspidati Rhizoma Et Radix),Niu Bang Zi(Great Burdock Fruit, Arctii Fructus),She Gan(Blackberry lily, Belamcandae Rhizoma),Jie Geng(Platycodon Grandiflorum, Platycodonis Radix),Chi Shao(red paeony root, Paeoniae Radix Rubra),Su Ye(Cultibated Purple Perilla Leaf, Perillae Folium),Jin Yin Hua(Honeysuckle Flower, Lonicerae Japonicae Flos),Shan Zha(Crataegus Pinnatifida, Crataegi Fructus),Gan Cao(Liquorice root, Glycyrrhizae	Zan 2023	SJ

		Radix Et Rhizoma)	
Healthy Qi — invigorating Anti-Inflammatory Formula	Decoction	Bai Zhu(Largehead Atractylodes Rh, Atractylodis Macrocephalae Rhizoma),Ban Lan Gen(Indigowoad Root, Isatidis Radix), Jin Yin Hua(Honeysuckle Flower, Lonicerae Japonicae Flos),Mian Ma Guan Zhong(Rhizoma dryopteris crassirhizomae, Dryopteridis Crassirhizomatis Rhizoma),Huo Xiang(Cablin Potchouli Herb, Pogostemonis Herba),Huang Qi(Milkvetch Root, Astragali Radix),Fang Feng(Divaricate Saposhniovia Root, Saposhnikoviae Radix),Jie Geng(Platycodon Grandiflorum, Platycodonis Radix),Sang Ye (Mulberry Leave, Mori Folium),Da Qing Ye(Indigowoad Leaf, Isatidis Folium),Gan Cao (Liquorice root, Glycyrrhizae Radix Et Rhizoma) ,Chai Hu(Chinese Thorowax Root, Bupleuri Radix),Ju Hua(Chrysanthemum flower, Chrysanthemi Flos)	Li JY 2020
Self-made Chinese medicinal formulae 1	Decoction	Guan Zhong (Japanese Flowering Fern Rhizome, Dryopteridis Crassirhizomatis Rhizoma),Jin Yin Hua (Honeysuckle Flower, Lonicerae Japonicae Flos),Lian Qiao (Weeping Forsythia Capsule, Forsythiae Fructus), Ban Lan Gen (Indigowoad Root, Isatidis Radix),Niu Bang Zi (Great Burdock Fruit, Arctii Fructus),Huo Xiang(Cablin Potchouli Herb, Pogostemonis Herba),Zhu Ye(Common Lophatherum Herb, Lophatheri Herba),Gan Cao (Liquorice root, Glycyrrhizae Radix Et Rhizoma), Da Qing Ye(Indigowoad Leaf, Isatidis Folium)	Liu BL 2010

Influenza Formulae	Preventive	Granule	Sang Ye (Mulberry Leave, Mori Folium), Xuan Fu Hua (Inula flower, Inulae Flos), Ku Xin Ren (Bitter Apricot Seed, Armeniacae Semen Amarum), Niu Bang Zi (Great Burdock Fruit, Arctii Fructus), Lian Qiao (Weeping Forsythia Capsule, Forsythiae Fructus), Fang Feng (Divaricate Saposhniovia Root, Saposhnikoviae Radix), Lu Gen (Phragmitis Rhizoma, Rhizoma Phragmitis)	Su JH 2024
