# Supplementary material for: Traditional Chinese Medicine for preventing influenza: a systematic review and meta-analysis
Source: Front Med (Lausanne). 2026 Apr 23;13:1736574. doi: 10.3389/fmed.2026.1736574 (PMC13149241; doi:10.3389/fmed.2026.1736574)
Supplement: Supplementary file 3 [file Data_Sheet_3.pdf]

Supplementary Materials 3

1.Newcastle-Ottawa Scale of cohort study

Bias domain	Selection				Comparability	Outcome			Total score
Signaling question	Representativeness of the exposed cohort	Selection of the non-exposed cohort	Ascertainment of exposure	Demonstration that outcome of interest was not present at start of study	Comparability of cohorts on the basis of the design or analysis controlled for confounders	Assessment of outcome	Was follow-up long enough for outcomes to occur	Adequacy of follow-up of cohorts	
Response options	a) Truly representative (one star)<br>b) Somewhat representative (one star)<br>c) Selected group<br>d) No description of the derivation of the cohort	a) Drawn from the same community as the exposed cohort (one star)<br>b) Drawn from a different source<br>c) No description of the derivation of the non exposed cohort	a) Secure record (e.g., surgical record) (one star)<br>b) Structured interview (one star)<br>c) Written self report<br>d) No description	a) Yes (one star)<br>b) No	a) The study controls for age, sex and marital status (one star)<br>b) Study controls for other factors (list) (one star)<br>c) Cohorts are not comparable on the basis of the design or analysis controlled for confounders	a) Independent blind assessment (one star)<br>b) Record linkage (one star)<br>c) Self reportd) No description	a) Yes (one star)<br>b) No	a) Complete follow up- all subject accounted for (one star)<br>b) Subjects lost to follow up unlikely to introduce bias- number lost less than or equal to 20% or description of those lostsuggested no different from those followed. (one star)<br>c) Follow up rate less than 80% and no description of those lost<br>d) No statement	

scores	0.5	1	1	1	2	1	1	1	8.5

2.ROBINS-I of non-randomized controlled trials

	Pre-intervention		At intervention	Post-intervention				
Study ID	Bias due to confounding	Bias in selection of participants into the study	Bias in classification of interventions	Bias due to deviations from intended interventions	Bias due to missing data	Bias in measurement of outcomes	Bias in selection of the reported result	Overall quality
Liu BL2010	Medium risk	Medium risk	Low risk	Low risk	Low risk	Low risk	Low risk	Medium risk
Luo Y 2013	Medium risk	Low risk	Low risk	Low risk	Low risk	Low risk	Low risk	Medium risk
Su JH 2024	Medium risk	Low risk	Low risk	Low risk	Low risk	Low risk	Low risk	Medium risk
