# Supplementary material for: Traditional Chinese Medicine for preventing influenza: a systematic review and meta-analysis
Source: Front Med (Lausanne). 2026 Apr 23;13:1736574. doi: 10.3389/fmed.2026.1736574 (PMC13149241; doi:10.3389/fmed.2026.1736574)

Outcome: The incidence of influenza (cohort study, TCM versus no treatment)

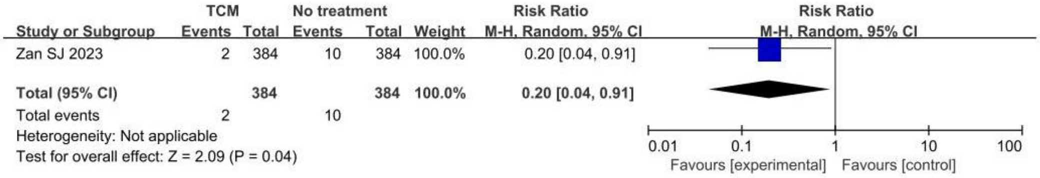

Outcome: The incidence of influenza-like illness (TCM versus no treatment, group by intervention)

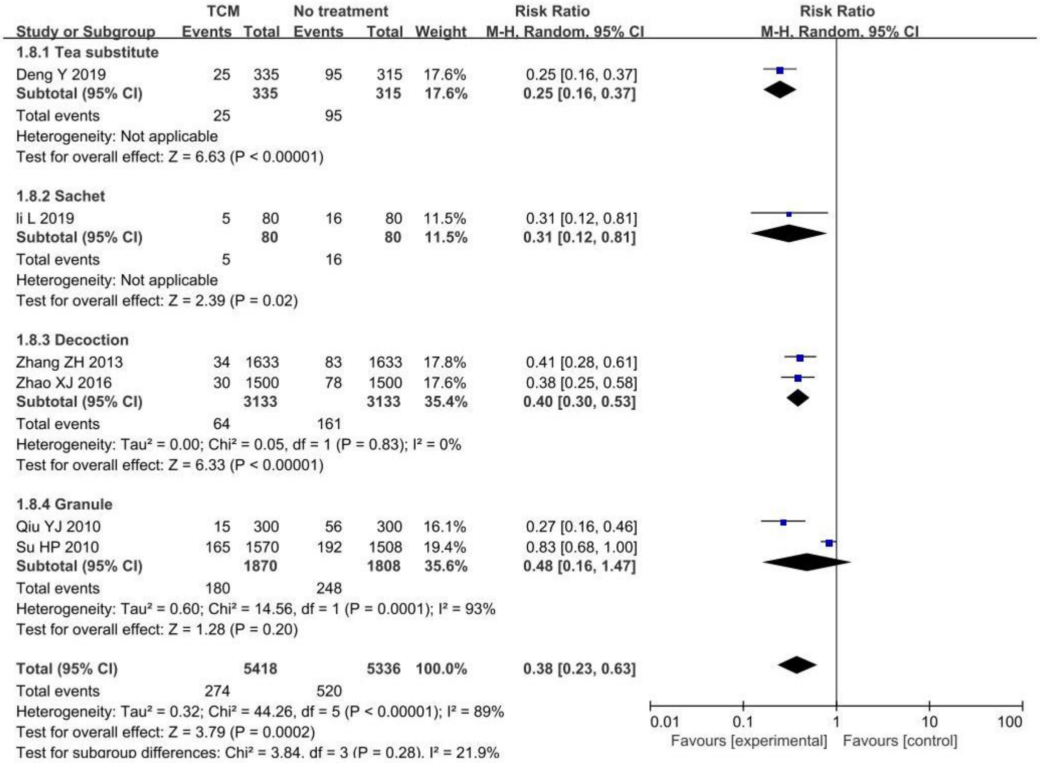

Outcome: The incidence of influenza-like illness (TCM versus no treatment, group by age)

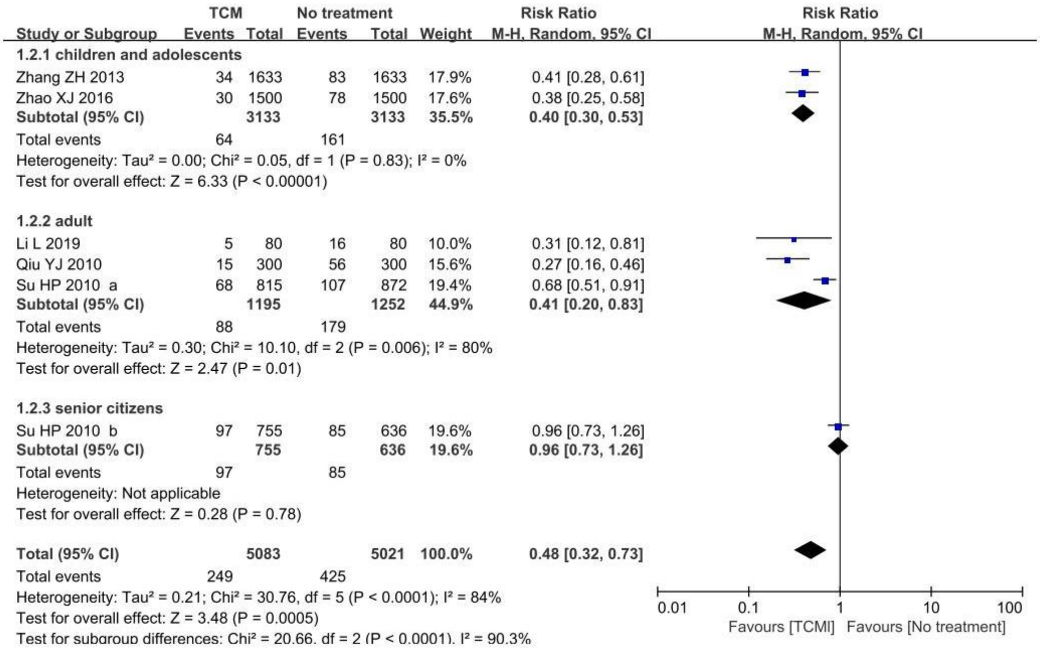

Outcome: Symptoms of influenza-like illness (TCM versus no treatment)

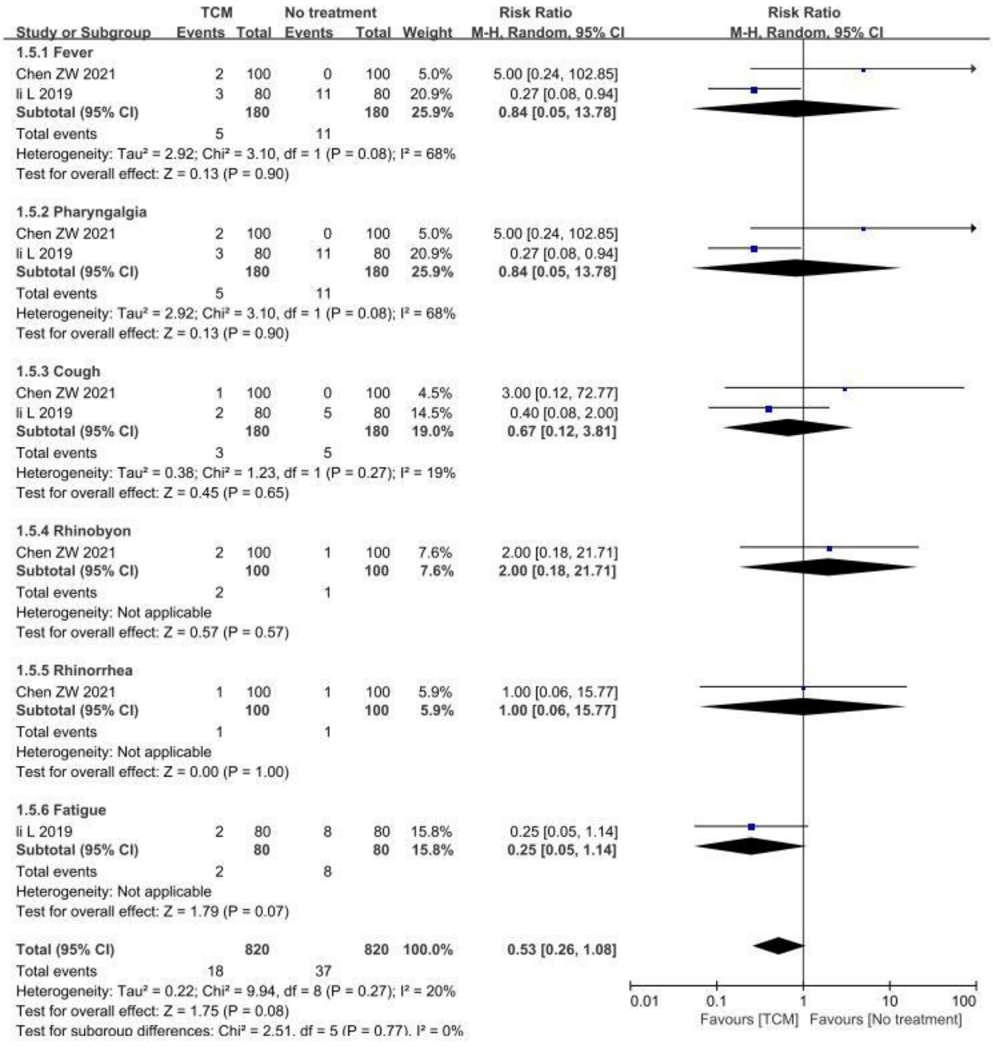

Outcome: Symptoms of influenza-like illness (TCM versus Indometacin)

	TCM		Other treatment			Risk Ratio
Study or Subgroup	Events	Total	Events	Total	Weight	M-H, Random, 95% CI
**1.6.1 Fever**						
Wang ZW 2008	10	45	18	40	23.7%	0.49 [0.26, 0.94]
**Subtotal (95% CI)**		**45**		**40**	**23.7%**	**0.49 [0.26, 0.94]**
Total events	10		18			
Heterogeneity: Not applicable						
Test for overall effect: Z = 2.14 (P = 0.03)						
**1.6.2 Chill**						
Wang ZW 2008	3	45	8	40	6.3%	0.33 [0.09, 1.17]
**Subtotal (95% CI)**		**45**		**40**	**6.3%**	**0.33 [0.09, 1.17]**
Total events	3		8			
Heterogeneity: Not applicable						
Test for overall effect: Z = 1.71 (P = 0.09)						
**1.6.3 Fatigue**						
Wang ZW 2008	10	45	20	40	25.0%	0.44 [0.24, 0.83]
**Subtotal (95% CI)**		**45**		**40**	**25.0%**	**0.44 [0.24, 0.83]**
Total events	10		20			
Heterogeneity: Not applicable						
Test for overall effect: Z = 2.53 (P = 0.01)						
**1.6.4 Headache**						
Wang ZW 2008	5	45	13	40	11.2%	0.34 [0.13, 0.87]
**Subtotal (95% CI)**		**45**		**40**	**11.2%**	**0.34 [0.13, 0.87]**
Total events	5		13			
Heterogeneity: Not applicable						
Test for overall effect: Z = 2.24 (P = 0.03)						
**1.6.5 Muscular soreness**						
Wang ZW 2008	9	45	19	40	22.1%	0.42 [0.22, 0.82]
**Subtotal (95% CI)**		**45**		**40**	**22.1%**	**0.42 [0.22, 0.82]**
Total events	9		19			
Heterogeneity: Not applicable						
Test for overall effect: Z = 2.53 (P = 0.01)						
**1.6.6 Anorexia**						
Wang ZW 2008	5	45	15	40	11.7%	0.30 [0.12, 0.74]
**Subtotal (95% CI)**		**45**		**40**	**11.7%**	**0.30 [0.12, 0.74]**
Total events	5		15			
Heterogeneity: Not applicable						
Test for overall effect: Z = 2.60 (P = 0.009)						
**Total (95% CI)**		**270**		**240**	**100.0%**	**0.41 [0.30, 0.56]**
Total events	42		93			
Heterogeneity: $Tau^2 = 0.00$; $Chi^2 = 1.13$, df = 5 (P = 0.95); $I^2 = 0\%$						
Test for overall effect: Z = 5.57 (P < 0.00001)						
Test for subgroup differences: $Chi^2 = 1.12$, df = 5 (P = 0.95), $I^2 = 0\%$						

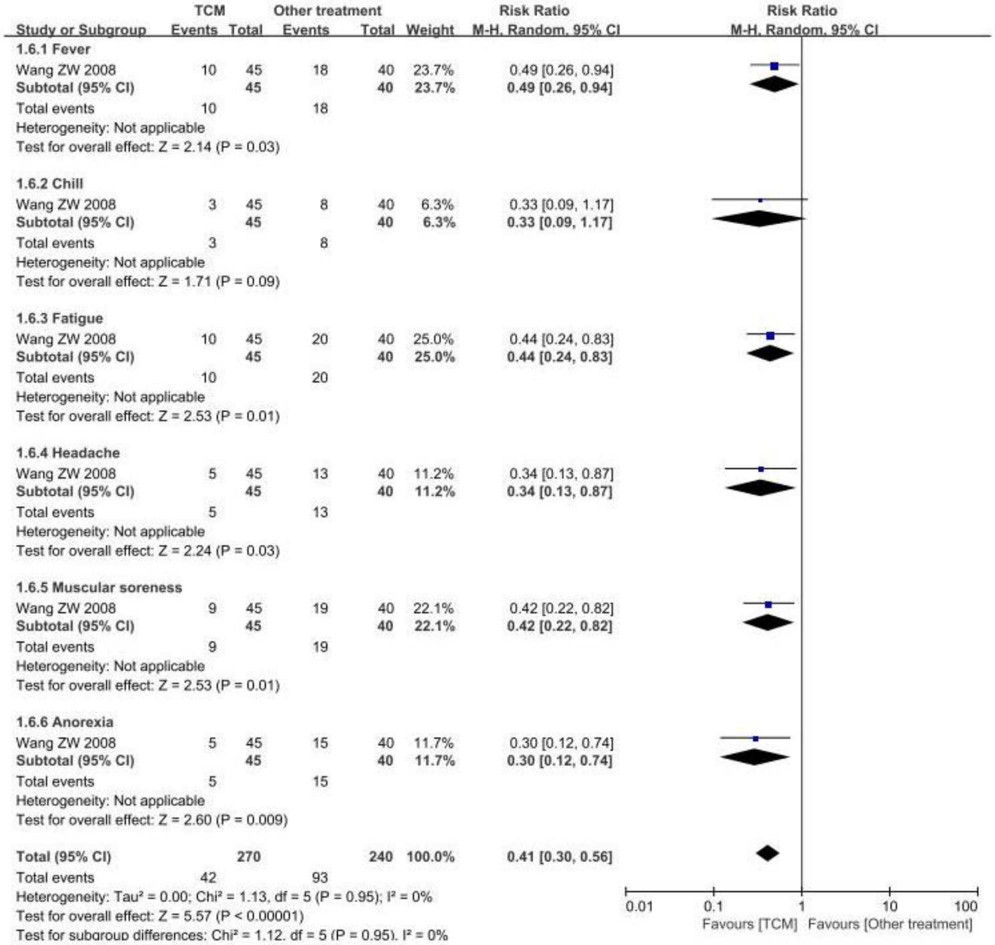

Outcome: Adverse events (cohort study, TCM versus no treatment)

	TCM		No treatment			Risk Ratio
Study or Subgroup	Events	Total	Events	Total	Weight	M-H, Random, 95% CI
Zan SJ 2023	9	384	0	384	100.0%	19.00 [1.11, 325.30]
**Total (95% CI)**		**384**		**384**	**100.0%**	**19.00 [1.11, 325.30]**
Total events	9		0			
Heterogeneity: Not applicable						
Test for overall effect: Z = 2.03 (P = 0.04)						

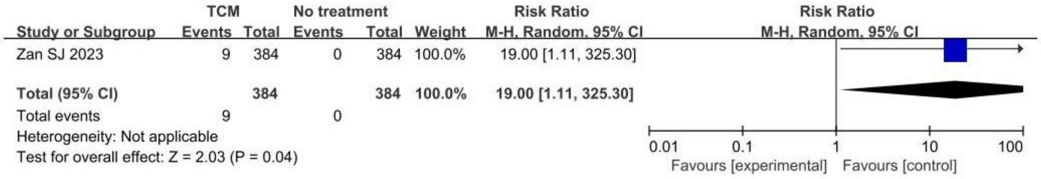

Outcome: Adverse events (TCM versus no treatment, group by age)

Study or Subgroup	TCM Events	TCM Total	No treatment Events	No treatment Total	Weight	Risk Ratio M-H, Random, 95% CI
**1.3.1 children and adolescents**						
Zhang ZH 2013	0	1633	0	1633		Not estimable
Zhao XJ 2016	0	1500	0	1500		Not estimable
**Subtotal (95% CI)**		**3133**		**3133**		**Not estimable**
Total events	0		0			
Heterogeneity: Not applicable						
Test for overall effect: Not applicable						
**1.3.2 adult**						
Chen ZW 2021	2	100	0	100	49.9%	5.00 [0.24, 102.85]
Deng Y 2019	0	335	0	315		Not estimable
Li JY 2020	0	105	0	105		Not estimable
Li L 2019	2	80	0	80	50.1%	5.00 [0.24, 102.53]
**Subtotal (95% CI)**		**620**		**600**	**100.0%**	**5.00 [0.59, 42.37]**
Total events	4		0			
Heterogeneity: Tau² = 0.00; Chi² = 0.00, df = 1 (P = 1.00); I² = 0%						
Test for overall effect: Z = 1.48 (P = 0.14)						
**Total (95% CI)**		**3753**		**3733**	**100.0%**	**5.00 [0.59, 42.37]**
Total events	4		0			
Heterogeneity: Tau² = 0.00; Chi² = 0.00, df = 1 (P = 1.00); I² = 0%						
Test for overall effect: Z = 1.48 (P = 0.14)						
Test for subgroup differences: Not applicable						

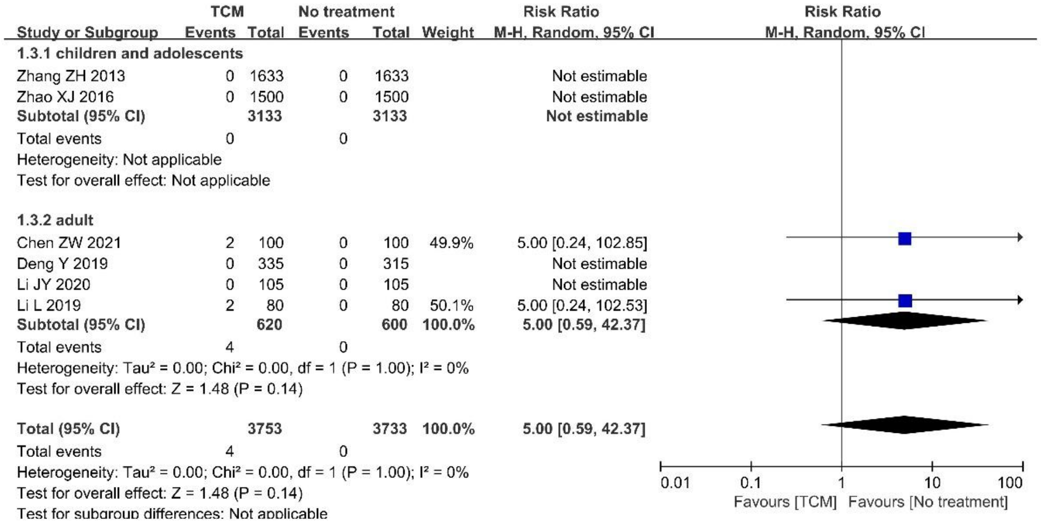

Outcome: Hospitalization rate (TCM versus no treatment)

Study or Subgroup	TCM Events	TCM Total	No treatment Events	No treatment Total	Weight	Risk Ratio M-H, Random, 95% CI
**1.3.1 Hospitalization rate**						
Deng Y 2019	0	335	0	315		Not estimable
**Subtotal (95% CI)**		**335**		**315**		**Not estimable**
Total events	0		0			
Heterogeneity: Not applicable						
Test for overall effect: Not applicable						
**1.3.2 Emergency (outpatient) attendance rate**						
Deng Y 2019	21	335	67	315	100.0%	0.29 [0.19, 0.47]
**Subtotal (95% CI)**		**335**		**315**	**100.0%**	**0.29 [0.19, 0.47]**
Total events	21		67			
Heterogeneity: Not applicable						
Test for overall effect: Z = 5.15 (P < 0.00001)						
**Total (95% CI)**		**670**		**630**	**100.0%**	**0.29 [0.19, 0.47]**
Total events	21		67			
Heterogeneity: Not applicable						
Test for overall effect: Z = 5.15 (P < 0.00001)						
Test for subgroup differences: Not applicable						

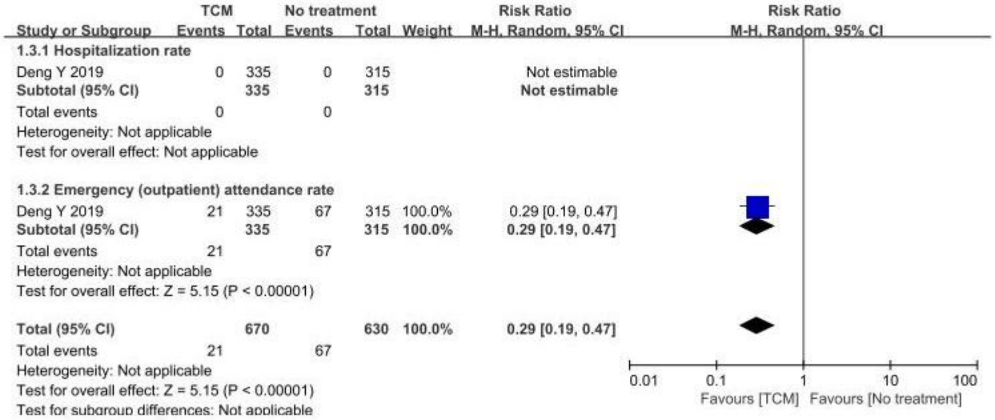

Supplement: Supplementary file 4 [file Data_Sheet_4.pdf]
